# A Systematic Research Review on Teachers’ Self-Efficacy in Educating Autistic Students

**DOI:** 10.1177/23969415251392318

**Published:** 2025-10-29

**Authors:** Linda Petersson-Bloom, Erika Hansson

**Affiliations:** 1 4342 Faculty of Education, Kristianstad University, SE-291 88 Kristianstad, Sweden; 2 4342 Center of Neurodevelopmental Disorders (KIND), Centre for Psychiatry Research, Department of Women's and Children's Health, Karolinska Institutet, Stockholm, Sweden

**Keywords:** education, autistic students, systematic research review, teachers’ self-efficacy

## Abstract

**Background and Aim:**

Ensuring equitable education for all students, including those with autism, is a core international commitment. The OECD (2018) defines equitable education as providing equal learning opportunities through responsive support tailored to individual needs. From this perspective, inclusive education involves both rights-based and needs-based approaches and emphasizes the importance of quality and adaptability in educational provision. Teachers’ self-efficacy—their perceived confidence and belief in their ability to teach and support autistic students—is a critical factor in achieving these aims. However, despite growing commitments to inclusion, challenges are frequently reported not only by teachers, but also by parents and autistic students, including feelings of exclusion, misunderstanding, and inadequate support within educational settings. This review aims to synthesize existing research on teachers’ self-efficacy in educating autistic students, identify influencing factors, assess methodological approaches, and outline future directions.

**Methods:**

A mixed-methods systematic review was conducted using a convergent integrated design. Qualitative, quantitative, and mixed-methods studies were synthesized. Searches were carried out in four databases: PsycInfo, ERIC, Education Source, and SCOPUS. Study quality was assessed using the Mixed Methods Appraisal Tool (MMAT; Hong et al., 2018), and the review followed PRISMA guidelines.

**Main Contribution:**

Fifty-seven studies were included. Findings indicate that although many teachers are willing to support autistic students, their self-efficacy is often undermined by limited professional development, structural barriers, and insufficient support. Key facilitators include sustained, practice-oriented professional development and supportive leadership. However, the predominance of self-report methods limits understanding of how self-efficacy translates into classroom practice.

**Conclusions:**

Teacher self-efficacy is shaped by both systemic conditions and individual perceptions of the ability to meet diverse student needs. Strengthening self-efficacy is essential to advancing equitable education for autistic students.

Despite decades of inclusive education reform, many autistic students continue to encounter barriers to participation, learning, and well-being across educational settings. This systematic review investigates the potential role of teachers’ self-efficacy in shaping how teachers perceive and respond to the needs of autistic students.

Schools play a critical role in children's overall development (Viner et al., 2012). Ensuring every student's right to equitable education and well-being is a foundational principle of international agreements, such as the UN Convention on the Rights of the Child (United Nations, 1989). Schools are expected not only to provide academic instruction but also to foster environments that promote student well-being. Over recent decades, national and international commitments to inclusive education have intensified. For instance, the Salamanca Statement (1994) advocated for the inclusion of students with special educational needs and disabilities (SEND) in general education. Subsequent frameworks, including the UN Convention on the Rights of Persons with Disabilities (United Nations, 2006) and General Comment No. 4 (2016), have further reinforced international obligations to inclusive education. Yet, implementing inclusive ideals remains difficult, especially for autistic students. Despite sustained policy support, school environments often pose significant challenges for students with neurodevelopmental disabilities (NDDs) such as autism, as well as for the educators who support them (Roberts & Webster, 2022). These persistent difficulties underscore the need for teachers to be adequately prepared to address the diverse educational needs of students with disabilities, including autism. Transitioning to the broader concept of equity in education, defining “equitable” practice is inherently complex. The Organization for Economic Co-operation and Development (OECD, 2018) defines equitable education as offering equal learning opportunities by addressing the varied needs and circumstances of all students, while ensuring appropriate support for their development and academic success. Based on this definition, and for the purpose of this review, we interpret equitable education not as endorsing a uniform model of placement, but as an approach that priorities student well-being, development, and access to learning in contexts most suitable to their individual needs. The focus, therefore, lies in the quality and responsiveness of educational provision, rather than its specific form. This interpretation is supported by research highlighting that autistic students experience improved well-being and learning outcomes in flexible, needs-responsive settings (Goodall, 2019).

From this viewpoint, inclusive education can be seen as an integrative concept encompassing both a rights-based perspective—emphasizing all students’ participation in general education—and a needs-based perspective—highlighting the necessity of tailored settings and supports responsive to individual needs (Ravet, 2011). These perspectives are not mutually exclusive; rather, they are complementary. This interpretation reflects a pedagogical stance focused on responsiveness, where students’ participation and well-being are supported through both their rights and their individual support needs (Ravet, 2011). Accordingly, our interpretation of equitable education centers on the quality and adaptability of provision, aiming to ensure participation, well-being, and development for each student in the most appropriate context.

Achieving equity in education, therefore, depends not only on structural policy but also on educators’ daily capacity and confidence to address diverse needs. Teacher self-efficacy can thus be understood as a key enabling factor for equitable education, representing teachers’ perceived capacity to respond flexibly and confidently to diverse student needs. For autistic students, numerous studies highlight persistent challenges in attaining a positive school experience. These difficulties are echoed by families and teachers, who frequently encounter barriers to effective inclusion (Petersson-Bloom & Holmqvist, 2022; Roberts & Webster, 2022). Research shows that autistic students face an elevated risk of school exclusion (Brede et al., 2017; Nordin et al., 2023). While the causes of exclusion vary, they are often linked to inadequacies within the learning environment, including sensory sensitivities, interpersonal conflicts, and the failure of schools and teachers to recognize and accommodate the distinct needs of autistic students (Brede et al., 2017; Nordin et al., 2023; Sproston et al., 2017).

Recent research continues to highlight how autistic students often are misunderstood, excluded, or inadequately supported in general education settings (Gray et al., 2023; Lynam et al., 2024). Gray et al. (2023) found that many autistic students experienced school as emotionally distressing and overwhelming, often leading to school avoidance or exclusion. Rather than framing this as individual refusal, the findings emphasize how negative experiences arise from a mismatch between student needs and the school environment. Parents and professionals in Gray et al. (2023) also described limited staff understanding, adversarial home-school relationships, and systemic barriers to accessing support. This phenomenon can also be described as “*school can’t*,” which frames attendance difficulties not as refusal but as an involuntary response to environments that are experienced as overwhelming or unsafe (Heimans et al., 2024). Similarly, Lynam et al. (2024), in a scoping review and consultation with autistic pupils, reported that feelings of being misunderstood, bullied, excluded, or overwhelmed were common across primary and postprimary settings. These findings underscore the need to go beyond structural supports and create school environments where autistic pupils feel heard, respected, and understood.

Atkinson et al. (2024) conducted a systematic review of qualitative studies on the experiences of autistic students in primary schools. While some students described positive relationships and learning experiences, many reported long-term negative effects on mental health. Students emphasized the importance of educators having a better understanding of autism and offering subtle, individualized support. Alongside these findings, Holmes (2022) reported that autistic students often encountered inconsistent teacher understanding of autism from year to year, contributing to confusion and disconnection. Participants noted a general lack of awareness and acceptance from both teachers and peers and called for more inclusion of autistic voices in research and educational decision-making.

A meta-analysis of teachers’ implicit attitudes revealed a moderate bias in favor of nonmarginalized student groups, suggesting that unconscious processes may influence teacher behavior in ways that hinder equitable educational practices (Pit-Ten Cate & Glock, 2019). Building on this, Hasson et al. (2022) found that general education systems were frequently under-resourced and ill-equipped to support autistic students, placing considerable strain on families and frontline SEND educators. Although the dedication of parents/carers and teachers contributed to a sense of support and hope, the study emphasized the need for systemic policy reform to achieve sustainable inclusion. Research focused on autism has shown that positive teacher attitudes and perceived competence are essential for effective inclusion and better educational outcomes (Park & Chitiyo, 2011). However, even with generally positive attitudes, many teachers report feeling underprepared to support autistic learners. A recent systematic review of teachers’ beliefs revealed that, although inclusion was valued, many expressed low confidence in teaching autistic students, citing limited autism-specific knowledge and inadequate support (Han & Cumming, 2024).

These challenges underscore the importance of teachers’ self-efficacy in supporting autistic students—a critical element in advancing equitable education. A teacher's attributes, targeted training, adequate classroom resources, and institutional support can all contribute to strengthening teachers’ enhance self-efficacy, highlighting the need to understand how these factors influence teachers’ confidence in educating autistic students. Although individual studies have explored aspects of teacher self-efficacy related to autistic students, to the best of our knowledge, no systematic review has yet synthesized this body of research. This gap limits understanding of how teachers’ confidence develops and how educational environments can be adapted to equitably support the participation, development, and well-being of autistic students. This review addresses these gaps by systematically analyzing the literature on teachers’ self-efficacy in educating autistic students, including its scope, influencing factors, methodological approaches, and areas that remain underexplored.

## Teachers’ Self-Efficacy

Self-efficacy refers to an individual's belief in their ability to meet challenges and demands in a given situation. The concept was introduced by Bandura (1977) as a core component of social cognitive learning theory. In professional settings, self-efficacy reflects a person's perception of their capacity to manage various work-related tasks and achieve goals (Bandura, 1997). In teaching, “teacher efficacy” or “teacher self-efficacy” denotes a teacher's confidence in planning, organizing, and delivering instruction to help students achieve educational objectives (Skaalvik & Skaalvik, 2007). Notably, research suggests that teachers with greater confidence in their instructional abilities tend to experience less stress and are more resilient against burnout (Schwarzer & Hallum, 2008). Conversely, earlier studies indicate that teachers of autistic students often experience heightened emotional exhaustion, increasing the risk of burnout (Jennett et al., 2003).

Teacher self-efficacy has been conceptualized in various ways, ranging from broad domains such as classroom management, instructional strategies, and student engagement (Tschannen-Moran & Hoy, 2001), to subject- or context-specific areas like teaching particular subjects or working in diverse school environments (Klassen et al., 2011). Although most research focuses on general teacher self-efficacy, some scholars argue that population-specific domains, such as teaching students with disabilities, require more tailored conceptual and measurement approaches (Kosko & Wilkins, 2009; Sharma et al., 2012).

In response to these concerns, instruments such as the Teacher Efficacy for Inclusive Practices scale have been developed to assess inclusive teaching efficacy more broadly (Sharma et al., 2012). However, population-specific tools may be necessary to accurately capture teachers’ confidence in educating particular student groups, including autistic learners. Evidence also suggests that teachers with higher self-efficacy are more likely to adopt inclusive practices and retain students with SEND in general classrooms (Kosko & Wilkins, 2009).

## Aim and Research Questions (RQs)

The overarching aim of this systematic review is to synthesize existing research on teachers’ self-efficacy in educating autistic students. Specifically, the review investigates what is currently known about teachers’ self-efficacy beliefs in teaching and supporting autistic students, identifies the individual, contextual, and systemic factors that influence self-efficacy, and examines the characterizes of the studies to study this construct. The following RQs are addressed:

RQ1: What is currently known about teachers’ self-efficacy in teaching and supporting autistic students across diverse educational contexts?
RQ2: Which factors influence teachers’ self-efficacy in educating autistic students?
RQ3: What characterizes studies investigating teachers’ self-efficacy in educating autistic students?
RQ4: What gaps in the literature have been identified regarding teachers’ self-efficacy in educating autistic students?

## Method

This systematic review was conducted as a mixed-methods systematic review, drawing on Stern et al. (2020), who propose that mixed-methods reviews offer a more comprehensive foundation to inform practice and policy by incorporating findings from quantitative, qualitative, and mixed-methods research. While conducting mixed-methods reviews involves a certain degree of complexity, the primary aim is to integrate or combine quantitative and qualitative data to achieve both breadth and depth of understanding that may confirm or challenge existing evidence (Stern et al., 2020). Various approaches can be applied, and in this review, we adopted a convergent and integrated approach. This approach involves combining data extracted from quantitative, qualitative, and mixed-methods studies (Stern et al., 2020). A key step in this process is transforming the data into a compatible format (Voils et al., 2009). Following Frantzen and Fetters (2015), we chose to transform quantitative data into qualitative form, allowing conversion into themes, categories, or narratives. This review also followed the Preferred Reporting Items for Systematic Reviews and Meta-Analyses (PRISMA) guidelines (Page et al., 2021). The quality of the studies was assessed using the Mixed Methods Appraisal Tool (MMAT), Version 18 (Hong et al., 2018). This review was not preregistered.

### Search Strategy

The process of identifying potential search terminology began in December 2024. Following Cooke et al. (2012), the Sample, Phenomenon of Interest, Design, Evaluation, Research type (SPIDER) tool was used to identify search terms, with related inclusion and exclusion criteria applied in the initial phase (Table 1). The SPIDER framework serves as an alternative to tools like PICO, particularly when incorporating qualitative and mixed-methods studies (Cooke et al., 2012). Both authors collaborated iteratively to develop effective search strategies, adjusting them until a satisfactory outcome was achieved. During these early stages, we conducted test searches and evaluated various search string combinations, comparing their outcomes. A university-based librarian was also consulted to provide input and further refine the process. The final searches were completed in February 2025.

**Table 1.** Sample, Phenomenon of Interest, Design, Evaluation, Research Type (SPIDER) Framework.

Sample	Teachers in primary, middle, and secondary school (BE)/elementary and high school (AE)
Phenomenon of interest	Teacher efficacy/self-efficacy teaching autistic students
Design	Questionnaires, interviews
Evaluation	Experiences
Research type	Quantitative/mixed methods/qualitative

Searches were conducted in four electronic indexing databases: PsycInfo, Education Resources Information Centre, Education Source, and SCOPUS. These databases were selected for their relevance to the review's aim and research questions. The following search terms were used: Block one—(“Teacher* effective*” OR “Teacher* efficacy” OR “Self-efficacy in teacher*” OR “Self-efficacy”); Block two—(“Autism” OR “Autism spectrum disorder*” OR “Asperger syndrome” OR “autistic” OR “ASD”). Records identified during the search were imported into the reference management software Rayyan (Ouzzani et al., 2016) to facilitate independent screening by both reviewers.

### Inclusion and Exclusion Criteria

Based on the aim, the SPIDER framework was implemented, and search strategies were developed alongside relevant inclusion and exclusion criteria. The terms self-efficacy and teacher efficacy were both included, but only in the context of autism. In contrast, studies focusing on self-efficacy and teacher efficacy related to other NDDs were excluded. Both general and special education teachers, across multiple school years and various placements (e.g., regular and special settings), were included. In line with the review's methodology, qualitative, quantitative, and mixed-methods research were eligible. To be included, records had to be original studies published in English in peer-reviewed journals between 2005 and 2025. The inclusion and exclusion criteria are illustrated in Table 2.

**Table 2.** Inclusion and Exclusion Criteria.

Inclusion	Exclusion
Studies on self-efficacy or teacher efficacy related to autism	Studies on self-efficacy or teacher efficacy related to NDD
General education teachers, special educators, and special education teachers	Parents and students
Primary, middle, and secondary education (K-12 in the UK and AE equivalents)	Preschool and higher education
All school settings (e.g., regular schools, special schools, and special classes)	
Qualitative, quantitative, and mixed-methods studies	
Peer-reviewed journal articles	Grey literature, books, conference proceedings, editorials, and dissertations
	Reviews and meta-analyses
Articles in English	
Years 2005–2025	

Abbreviations: NDDs = neurodevelopmental disabilities; AE = American English.

### Screening Process

**Figure 1.**
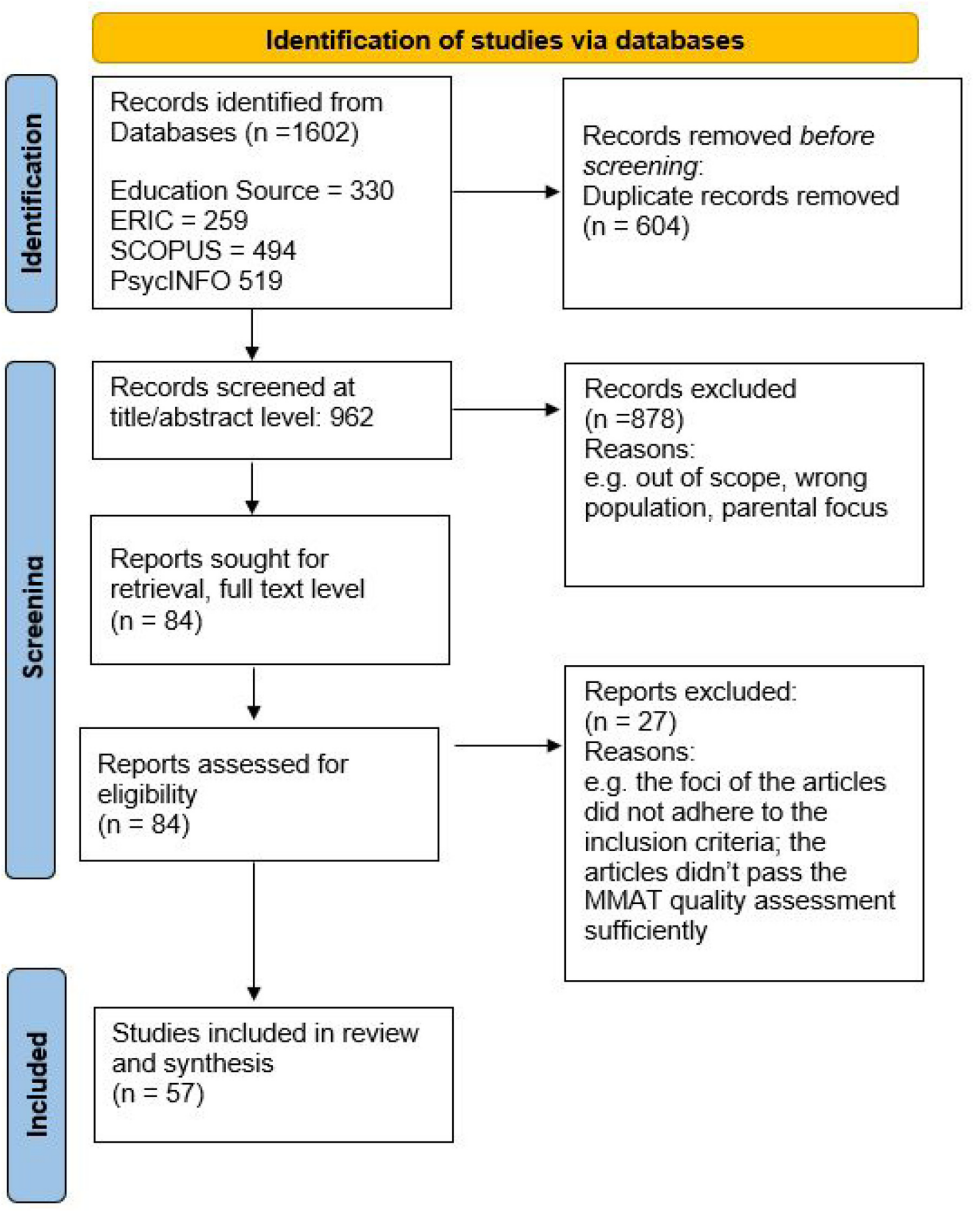
Preferred Reporting Items for Systematic Reviews and Meta-Analyses (PRISMA) 2020 Flow Diagram*. Note*. This figure was adapted from Page et al. (2021).

Initially, 1,602 articles were identified through database searches. A rigorous screening process was adopted, beginning with the import of all identified records into Rayyan software, where duplicates were removed. Next, 962 articles were screened at the title and abstract level independently by both authors, using Rayyan's blinded function to ensure objective review. The screening followed pre-defined inclusion and exclusion criteria (see Table 2). Inter-rater agreement was assessed using Cohen's kappa, which indicated substantial agreement between the reviewers (κ = 0.67). At this stage, 878 articles were excluded, primarily for being out of scope (e.g., not focusing on teachers’ self-efficacy) or involving an ineligible population (e.g., not including teachers). Disagreements were resolved through discussion based on the inclusion and exclusion criteria, leading to a unified decision and confirmation of eligibility. Subsequently, 84 full-text articles were reviewed by both authors in a combined step involving screening, data extraction, and quality assessment (see Figure 1).

### Study Selection, Data Extraction, and Quality Appraisal

In this step, all 84 articles were read in full, and a parallel process was implemented in which data were extracted and each study was assessed for quality. The two reviewers independently extracted data from and evaluated the quality of 42 articles each. To ensure consistency and inter-rater reliability, a 10% cross-check was conducted: each reviewer independently screened, extracted data from, and assessed the quality of four to five articles initially reviewed by the other. Following Kraus et al. (2020), a critical step in conducting a systematic review is to develop a data extraction sheet at the outset. Accordingly, a specific data extraction matrix was created by the first author prior to screening, aligned with the review's aim and research questions. Using a customized or adapted standardized extraction form is widely recommended (Büchter et al., 2020), along with piloting the data extraction sheet. The matrix used in this study was based on formats from prior systematic reviews (e.g., Petersson-Bloom & Holmqvist, 2022; Petersson-Bloom et al., 2025) and had thus been previously piloted.

The data extraction matrix included the following elements: author/year; country; type of school placement (i.e., general school, small class/resourced provision, or special school); participants; school level (i.e., primary or secondary); data collection method/type of scale; research design; research focus/aim; and findings/conclusions. Although data extraction and quality assessment were conducted in parallel, we agreed in advance that if a study's focus did not meet the inclusion criteria during data extraction, quality assessment would not proceed. For mixed-methods studies, we distinguished between those employing a specific mixed-methods design (e.g., convergent design; see Creswell & Creswell, 2018) and those using both qualitative and quantitative methods without a defined mixed-methods structure.

The data extraction matrix is presented in the Supplemental Material, data extraction matrix included studies. In addition to this article, a separate manuscript based on the same review process and dataset has been developed. That article focuses specifically on the psychometric properties and validity of the measurement instruments used to assess teacher self-efficacy in the included studies. While both articles draw from the same core data, they differ in aim, RQs, and analytical focus.

#### Quality Assessment

Given the inclusion of studies using qualitative, quantitative, and mixed-methods designs, methodological quality was assessed using the MMAT (Hong et al., 2018). A customized assessment form, developed by the first author based on the original MMAT framework and previously used in other studies (Petersson-Bloom et al., 2023, 2025), was employed for this purpose. The MMAT is specifically designed to evaluate the quality of empirical studies in systematic reviews that incorporate diverse methodological approaches. It includes five design-specific criteria for each study type—qualitative, randomized controlled trials, nonrandomized quantitative studies, descriptive quantitative studies, and mixed methods—along with two initial screening questions applicable to all research designs.

Studies were classified as mixed methods only if they met the definition provided by Hong et al. (2018). Assessors responded to each methodological criterion using one of three codes: yes (coded as A), no (coded as B), or cannot tell (coded as C). The two initial screening questions addressed the clarity of RQs (S1) and whether the collected data allowed for addressing those questions (S2). Following Hong et al. (2018), if either screening question was answered “no” or “cannot tell,” further assessment was not conducted. However, some studies lacked explicit research questions but presented a clearly defined aim. In such cases, we recorded “no” for S1 but noted the study's precise aim and included it in the assessment. In line with Hong et al. (2018), all studies that met the two initial screening criteria were retained for synthesis, regardless of their final methodological score. The complete results of the quality assessment are available in the Supplemental Material (Quality Assessment, MMAT).

### Finalized Study Selection

The cross-checking process revealed full agreement between reviewers, indicating strong consistency in data extraction and quality assessment judgments. Following this process, 57 studies were included in the final synthesis (see Figure 1).

#### Method for Synthesis

Following Frantzen and Fetters (2015), Heyvaert et al. (2017), and Stern et al. (2020), we employed a narrative synthesis approach, structured around the identification and interpretation of thematic categories. Consistent with recommended integrative review techniques for a convergent integrated approach (Stern et al., 2020), we qualitatively analyzed quantitative findings, converting numerical results into textual descriptions that could be meaningfully compared with qualitative data. Through repeated, detailed examination of all textual data, we identified patterns of meaning that formed the basis for the analytical categories. Finally, a thematic synthesis was conducted across the five categories. This synthesis drew on data extracted for the aspects of *Research focus*/*aim* and *Findings*/*conclusions* (see Supplemental Material: *Data Extraction Matrix, Included Studies*). The first author identified the thematic analytical categories and themes, which were then validated by the second author. These thematic categories and themes represent the primary outcome of the review and were derived through interpretative synthesis of findings across study types. As such, the aim was not to aggregate quantitative metrics but to understand patterns in how teachers’ self-efficacy has been explored and described.

## Results

The Characteristics of Included Studies section of the results presents the characteristics of the included studies, while the Analytical Categories of Included Studies section provides a narrative synthesis of the identified analytical categories and overarching themes.

### Characteristics of Included Studies

Of the 57 articles that met the inclusion criteria, 28 were published between 2020 and 2024. In the preceding period, from 2015 to 2019, 20 studies were published, while nine were identified from 2008 to 2014. No studies published before 2008 were found (see Supplemental Material: Data Extraction Matrix, Included Studies for detailed study characteristics). Most included studies were conducted in the United States (k = 16; e.g., Accardo & Finnegan, 2017; Catalano et al., 2023; Maddox & Marvin, 2013), followed by the United Kingdom—including both Scotland and England (k = 7; e.g., Alallawi et al., 2022; Humphrey & Symes, 2013; Wearmouth & Butler, 2020)—and Ireland (k = 6; e.g., Rodden et al., 2019; Ryan & Mathews, 2022a). A total of 25 studies were conducted in various European countries, including France (k = 1; Boujut et al., 2017), Slovakia (k = 1; Nemček et al., 2024), and Spain (k = 2; Latorre-Cosculluela et al., 2022, 2023), among others. Six studies were conducted in Asian countries, such as South Korea (k = 1; Park et al., 2019), Malaysia (k = 1; Selvaganapathi et al., 2019), and China (k = 2; Lu et al., 2020; Xie et al., 2024). A few studies were conducted across multiple countries, including collaborations between France and Canada (k = 1; Cappe et al., 2021), Croatia, North Macedonia, and Poland (k = 1; Lisak Šegota et al., 2022), and the United States and Australia (k = 1; Love et al., 2019).

Regarding school settings, most studies (k = 28; e.g., Latorre-Cosculluela et al., 2023; Rodden et al., 2019; Wittwer et al., 2024) were conducted in general education settings. This was followed by studies conducted across multiple settings (k = 11; e.g., Baek et al., 2024; Cappe et al., 2021; Lisak Šegota et al., 2022), smaller or resourced classes (k = 6; e.g., Love et al., 2020; Ryan & Mathews, 2022a), and special schools (k = 5; e.g., Breeman et al., 2016; Park et al., 2019). In seven studies, the school setting was not explicitly reported. In terms of school level, studies were most frequently conducted in primary school settings (k = 23; e.g., Bitsika et al., 2017; Johnson et al., 2021; Snyman et al., 2023), while an equal number (k = 24) spanned multiple school years (e.g., Love et al., 2019; Nolan & Hannah, 2019; Siu & Ho, 2010). Only three studies were conducted exclusively in secondary schools (e.g., Humphrey & Symes, 2013; Nemček et al., 2024; Rodden et al., 2019), and seven studies did not specify the school level.

Most included studies (k = 41) employed a quantitative research design, primarily using questionnaires for data collection. Only seven studies used a qualitative design, typically involving interviews (mostly semi-structured). Some studies incorporated multiple data collection methods (e.g., Catalano et al., 2023; Maddox & Marvin, 2013), while just three employed a mixed-methods design (k = 3; Horan & Merrigan, 2019; Nolan & Hannah, 2019; Park et al., 2019).

### Result of Quality Assessment

The results of the quality assessment for the included studies are fully presented in the Supplemental Material (Table: Quality Assessment, MMAT). Nine studies lacked an explicit research question; however, each had a clearly defined aim, allowing both initial assessment questions—Are there clear research questions? (S1) and Do the collected data allow addressing the research questions? (S2)—to be evaluated (see Quality Assessment, MMAT, Supplemental Material). In seven of the included studies—all classified as qualitative—the assessors answered “yes” (A to all design-specific quality criteria [Anglim et al., 2017; Boujut et al., 2017; Cook & Ogden, 2021; Devi & Ganguly, 2024; Rodden et al., 2019; Wearmouth & Butler, 2020; Zappalà & Aiello, 2023]). For the remaining 50 studies, quality assessments varied. For instance, some randomized controlled trials (RCTs) lacked sufficient information regarding whether outcome assessors were blinded to the intervention. Among the studies categorized as quantitative non-randomized, several did not clearly report confounders or failed to demonstrate that participants were representative of the target population. In the group of quantitative descriptive studies, several were assessed as using unrepresentative samples and failing to report adequately on the risk of non-response bias.

### Analytical Categories of Included Studies

Five analytical categories were developed based on patterns identified across the included studies: (1) general profiles and patterns of teacher self-efficacy, (2) professional development and targeted training in relation to teacher self-efficacy, (3) teachers’ self-efficacy related to inclusion and participation, (4) self-efficacy, stress, and burnout, and (5) development of self-efficacy scales. A detailed key to the analytical categories is presented in the Supplemental Material. The thematic synthesis conducted across these five categories led to the identification of two overarching themes: (1) factors that facilitate teacher self-efficacy and (2) factors that hinder teacher self-efficacy. These themes are further explored in the discussion.

#### Category 1: General Profiles and Patterns of Teacher Self-Efficacy

This category includes 22 studies focused on describing and/or measuring teachers’ self-efficacy in relation to autistic students. The term “general profiles and patterns” refers here to studies that broadly explore teacher self-efficacy in relation to instructional strategies, autism-related knowledge, or educational settings, without a targeted intervention or training component. For details on country and school context, see the section “Characteristics of Included Studies.”

This category encompasses studies examining teacher self-efficacy across various countries (e.g., Baek et al., 2024; Cappe et al., 2021; Kingsdorf et al., 2024; Lisak Šegota et al., 2022). For example, Baek et al. (2024) examined teachers’ willingness to provide accommodations and their self-efficacy in teaching autistic students. Although teachers expressed willingness to make accommodations, they lacked confidence in teaching this group effectively. The category also includes studies that integrate related dimensions such as attitudes and knowledge about autism and evidence-based practices (e.g., Rakap et al., 2018; Selvaganapathi et al., 2019; Stošić et al., 2022; Wearmouth & Butler, 2020; Wittwer et al., 2024).

Some studies examined differences in teacher self-efficacy based on educational placement—whether students were taught in general or special classes (e.g., Cook & Ogden, 2021; Ryan & Mathews, 2022b). Cook and Ogden (2021) found that differences in teacher self-efficacy across school settings reflected contrasting structural and cultural conditions. Teachers in special schools expressed greater confidence, supported by specialist training and resources, whereas general educators, despite valuing inclusion, often reported reduced self-efficacy due to systemic constraints rather than student characteristics.

Additionally, this category includes studies focusing on didactical or instructional strategies. For instance, Accardo and Finnegan (2017) investigated predictors of teachers’ perceived self-efficacy in teaching reading comprehension. Their results indicated that only 5% of teachers reported high levels of self-efficacy in this area.

Most studies indicate that teachers face challenges with their self-efficacy when teaching autistic students. An exception is found by Wearmouth and Butler (2020), who examined Special Educational Needs Coordinators (SENCOs). Their findings suggest that while SENCOs expressed confidence in their own abilities, they lacked confidence in the abilities of general classroom teachers.

#### Category 2: Professional Development and Targeted Training in Relation to Teacher Self-Efficacy

Nineteen studies focused on professional development or teacher training. This category includes research examining teachers’ participation in professional development and whether such participation is associated with self-efficacy. The studies reported a wide range of professional development efforts, varying in content and duration. All studies indicated an increase or positive change in teachers’ self-efficacy following participation in training sessions or development initiatives. These efforts aimed to enhance teachers’ knowledge of how to teach and support autistic students in classroom settings (e.g., Horan & Merrigan, 2019; Johnson et al., 2021; Nolan & Hannah, 2019; Rakap et al., 2015) or to combine autism-related content with specific areas of practice. For instance, Corona et al. (2017) examined autism training delivered alongside positive behavior support, while Öhlböck et al. (2024) investigated the effects of training using the Zones of Regulation curriculum on teachers’ self-efficacy. Three studies (Bitsika et al., 2017; Breeman et al., 2016; Park et al., 2019) focused specifically on teachers’ self-efficacy in managing student behavior, particularly in understanding or preventing challenging behaviors. For example, Bitsika et al. (2017) implemented a training session on functional behavior assessment to evaluate changes in teachers’ self-efficacy and confidence. Although some studies reported effect sizes or statistical outcomes, these were only noted during data extraction and were not included in the synthesis, as the aim was to identify thematic patterns rather than compare quantitative effects.

#### Category 3: Teachers’ Self-Efficacy Related to Inclusion and Participation

While conceptually related to Category 1, this category comprises a distinct group of studies focused specifically on teachers’ self-efficacy in the context of inclusive education and the participation of autistic students. Although some thematic overlap exists, studies in this category were selected for their explicit emphasis on inclusive practices, student participation, and educational decision-making. Eight studies were identified within this category, all of which examined teachers’ self-efficacy related to inclusion and participation.

Several studies explored general aspects of teacher self-efficacy in inclusive contexts (e.g., Devi & Ganguly, 2024; Humphrey & Symes, 2013; Latorre-Cosculluela et al., 2022, 2023). Findings from Latorre-Cosculluela et al. (2022, 2023) suggest that teacher efficacy and trust in their professional practice positively influence attitudes toward inclusion. Alhumaid (2021) and Nemček et al. (2024) focused specifically on physical education teachers’ self-efficacy regarding the inclusion of autistic students, both reporting moderate levels of self-efficacy. Alhumaid (2021) further noted that strong academic preparation positively impacts teacher self-efficacy.

Xie et al. (2024) adopted a broader perspective, examining how three school-level factors—administrative support, emotional support, and transformational leadership—along with in-service training, influenced teachers’ attitudes toward inclusive education. The study found that self-efficacy played a mediating role in these relationships. Lastly, Alkeraida (2023) analyzed teachers’ decision-making regarding the participation of autistic students, reporting that teachers with negative attitudes and lower self-efficacy made minimal efforts to promote participation.

#### Category 4: Self-Efficacy, Stress, and Burnout

This category pertains to teachers’ self-efficacy in relation to internal emotional experiences, specifically stress, burnout, and emotional coping mechanisms. This theme was identified in four studies. Boujut et al. (2017) examined the relationship between perceived stress, coping strategies, and self-efficacy, finding that lower levels of self-efficacy were associated with greater reliance on emotion-focused coping strategies, which increased the risk of burnout. Teachers with low self-efficacy were more likely to perceive stressful situations as threats or losses, contributing to emotional exhaustion. The study also indicated that coping strategies and perceptions of inclusion directly influence teachers’ adjustment to professional demands, and that self-efficacy plays a protective role in mental health.

Cappe et al. (2017) found that teachers of autistic students did not exhibit higher burnout levels than those teaching neurotypical students. However, in specialized settings, burnout was predicted by self-efficacy, whereas in general classrooms, perceived stress and social support were key predictors. Love et al. (2020) reported that higher teacher self-efficacy was associated with more positive engagement with autistic students, which in turn correlated with better student outcomes, including greater attainment of IEP goals. Finally, Ruble et al. (2011) identified significant associations between teachers’ physiological and affective states and their self-efficacy beliefs, although no associations were found for other sources of self-efficacy. Although some studies reported correlation coefficients, these were not consistently extracted or compared, in line with our thematic synthesis approach, which focused on narrative interpretation rather than statistical strength.

#### Category 5: Development and Validation of Self-Efficacy Scales

Four studies focused on developing and validating instruments to measure teachers’ self-efficacy in teaching autistic students. Catalano et al. (2023) developed the *Teacher Self-Efficacy for Teaching Students with ASD in Inclusive Early Childhood Classrooms Scale* (TSE-ASDI/EC), a 16-item scale shown to be highly reliable and specifically designed for early childhood settings. Love et al. (2019) introduced the *Teacher Self-Efficacy for Students with Autism Scale* (TSEAS), emphasizing the importance of using student-specific tools to assess self-efficacy for this population. Oh and Kozub (2010) developed the *Difficult Behavior Self-Efficacy Scale*, which demonstrated factorial validity and reliability across samples from Korea and the United States. Finally, Ruble et al. (2013) evaluated the *Autism Self-Efficacy Scale for Teachers*, providing preliminary support for its reliability and one-dimensionality, with expected correlations with stress but weak or absent correlations with general burnout. While psychometric properties such as reliability and factorial structure were sometimes reported, this review did not evaluate them systematically. These aspects are addressed in a companion article focusing specifically on measurement issues (see p. 15).

## Discussion

This systematic review aimed to synthesize research on teachers’ self-efficacy in educating autistic students by examining existing knowledge on their self-efficacy beliefs, the factors influencing these beliefs, and what characterizes these studies. Overall, the findings reveal persistent challenges in teacher self-efficacy related to educating autistic students—a pattern consistent with previous research on teacher preparedness and system-level support (Han & Cumming, 2024; Hasson et al., 2022), as well as with studies investigating autistic students’ experiences in school contexts (Atkinson et al., 2024; Gray et al., 2023; Lynam et al., 2024). Drawing from patterns across the five analytical categories, two overarching themes were identified: factors that facilitate teacher self-efficacy and factors that hinder it. These themes offer a broader lens for interpreting the findings, providing insight into both practical implications and future research directions. They also highlight the central role of teacher self-efficacy in advancing responsive and equitable education for autistic students.

### What Facilitates Teacher Self-Efficacy

Several studies in this review identified factors that strengthen teachers’ self-efficacy in educating autistic students. A key facilitator is participation in professional development, particularly when sustained, practice-oriented, and specifically tailored to teaching autistic learners. Such training was associated with improved understanding of autism and increased confidence in adapting instruction, managing challenging behavior, providing accommodations, and supporting student participation (e.g., Alhumaid, 2021; Breeman et al., 2016; Horan & Merrigan, 2019; Nolan & Hannah, 2019). This aligns with earlier research (Petersson-Bloom et al., 2023), which underscores the essential role of professional development in supporting equitable education by enhancing teachers’ ability to meet the diverse needs of autistic students.

In line with the equity framework outlined in the introductory part, these findings highlight that strengthening teacher self-efficacy through targeted professional learning is central to creating responsive educational environments that foster participation, development, and well-being. However, few studies provided details about the type of professional development, including whether it was neurodiversity affirming, evidence-based, or informed by lived experience. This lack of detail limits our understanding of how different types of professional development may differentially influence teacher self-efficacy. Similar concerns have been raised in earlier studies. Petersson-Bloom et al. (2023) concluded that while professional development may strengthen teachers’ confidence and knowledge, the content and impact of such efforts are often underreported and inconsistently implemented. In a related study, Petersson-Bloom and Holmqvist (2024) found that although teachers understanding improved, students and parents did not always experience notable changes in classroom practice, highlighting the need for deeper, more sustained support structures. Nevertheless, professional development embedded in daily practice appears especially important for achieving lasting improvements in teacher self-efficacy. However, variation in training content and delivery across studies indicates a need for further research to determine which models are most effective and sustainable. While professional development is a key driver, broader contextual factors—such as institutional support, leadership engagement, and access to specialist resources—also contribute significantly (e.g., Accardo & Finnegan, 2017; Baek et al., 2024; Cook & Ogden, 2021; Egan & Kenny, 2022; Xie et al., 2024). These systemic elements are not merely logistical supports; they are foundational to achieving equitable education, enabling teachers to respond confidently and adaptively in complex classroom settings. Furthermore, consistent with prior research (Roberts & Webster, 2022), this review reveals an ongoing gap between equity-based policy commitments and the practical realities teachers face. The findings suggest that realizing equitable education in practice requires not only professional skill development but also structural and cultural conditions that sustain inclusive and equitable efforts.

### What Hinders Teacher Self-Efficacy

The barriers identified in this review were not merely the absence of facilitators but rather a combination of structural, emotional, and perceptual factors that actively interfered with teachers’ self-efficacy. A prominent barrier across the reviewed studies was limited autism knowledge and inadequate practical preparation. General education teachers were frequently described as lacking sufficient training, feeling uncertain about instructional adaptations, and struggling to manage challenging situations (e.g., Lisak Šegota et al., 2022; Rakap et al., 2018; Rodden et al., 2019). These findings align with previous research indicating that, despite positive attitudes, many teachers feel underprepared to meet the needs of autistic students (Han & Cumming, 2024; Park & Chitiyo, 2011).

Studies also highlighted structural and contextual constraints, including limited planning time, inadequate staffing, and insufficient access to specialist support, which restricted the implementation of inclusive practices (e.g., Cook & Ogden, 2021; Wittwer et al., 2024). These challenges reflect broader concerns in the literature regarding the gap between inclusive policy ideals and the realities within general education settings (Hasson et al., 2022; Roberts & Webster, 2022). Emotional strain and burnout further emerged as significant barriers in studies exploring the relationship between self-efficacy, stress, and teacher well-being. Low self-efficacy was often linked to increased emotional exhaustion, particularly in general education contexts with limited institutional and peer support (Boujut et al., 2017; Cappe et al., 2017). These results are consistent with earlier research on the emotional cost of unsupported inclusion efforts (Jennett et al., 2003).

Some studies also described how teachers perceived the needs of autistic students as inherently overwhelming or unmanageable. In such cases, challenges were attributed to student characteristics rather than to modifiable instructional or environmental factors, reinforcing deficit-oriented thinking (Cook & Ogden, 2021). This aligns with Holmes (2022), who, drawing on students’ perspectives, reported that inconsistent teacher understanding of autism contributed to exclusion and disconnection. Collectively, these findings suggest that teacher self-efficacy is shaped not only by external supports but also by how challenges are framed and understood. Addressing both practical limitations and teachers’ perceptions is essential to strengthening self-efficacy and achieving equitable, inclusive education.

### Limitations

This review has several limitations. First, the selected inclusion and exclusion criteria may have led to the omission of relevant studies, resulting in an unintentionally biased sample. Second, restricting the review to English-language publications and excluding grey literature may have excluded important findings. Third, the methodological heterogeneity of the included studies may have influenced the synthesis. However, this heterogeneity also allowed for the inclusion of valuable insights from diverse disciplines and research traditions. Fourth, although quality assessment was conducted, we followed Hong et al. (2018) in opting not to exclude studies solely based on lower quality. While this approach supported broader inclusion, it may have affected the overall validity of the review. Fifth, although the search strategy was carefully constructed around the self-efficacy construct, studies that used broader or alternative terminology (e.g., “self-confidence” or “perceived competence”) may not have been captured. These terms were intentionally excluded to maintain conceptual precision in line with Bandura's (1977, 1997) definition of self-efficacy. While this decision preserved conceptual clarity, it may also have limited the scope of the included literature.

Despite these limitations, this review offers a comprehensive synthesis of the literature on teachers’ self-efficacy in educating autistic students. By incorporating studies from varied educational contexts and methodological traditions, it provides a nuanced understanding of the factors influencing teacher self-efficacy. The systematic approach—including transparent screening, quality assessment, and narrative synthesis—strengthens the credibility and relevance of the findings for both research and practice.

### Future Research

Several critical research gaps were identified in this review, which may guide future research. Most included studies were conducted in Western countries and focused on general education settings, particularly at the primary level. Consequently, secondary school contexts, special schools, and smaller provisions remain underrepresented. This is notable given prior findings that autistic students often experience inconsistent teacher understanding from year to year (Holmes, 2022). Future studies should explore how teachers’ self-efficacy—understood as their perceived confidence and competence in supporting autistic students—manifests and develops across varied educational settings. This includes examining how context-specific demands, such as academic pressures in secondary schools or resource limitations in specialist settings, influence teachers’ beliefs in their capacity to support autistic students. This review also identified a need for greater transparency in how professional development is designed, delivered, and framed. Future research should therefore investigate not only content and delivery models, but also include the perspectives of both teachers and students. This aligns with prior work that has similarly emphasized the importance of inclusive, well-documented professional development approaches (Petersson-Bloom et al., 2023; Petersson-Bloom & Holmqvist, 2024).

Additionally, the predominance of quantitative self-report methods limits understanding of how self-efficacy translates into effective instructional practice and meaningful relational engagement. While these measures reveal patterns in perceived confidence, they offer limited insight into how self-efficacy is enacted during day-to-day teaching. Future research would benefit from qualitative, observational, or mixed-methods designs to investigate how teachers’ self-efficacy evolves in real-time interactions with students, its impact on instructional strategies, and its relationship to both teacher development and student outcomes over time. Another important direction involves including autistic students in evaluating how teacher self-efficacy is expressed in classroom settings. Such perspectives could offer deeper insight into how teachers’ confidence and instructional decisions are perceived and experienced by students—an area emphasized in previous research (Fayette & Bond, 2018; Lynam et al., 2024; Petersson-Bloom et al., 2025; Zanuttini, 2023).

### Implications for Practice

The findings of this review highlight several practical priorities for enhancing teachers’ self-efficacy in educating autistic students. Sustained, autism-specific professional development should be embedded in both initial teacher education and ongoing professional learning, with a focus on applying knowledge in classroom contexts. It is equally important to foster school cultures that build and reinforce teacher confidence, rather than merely emphasizing compliance with policy requirements. Addressing structural barriers—such as limited planning time, inadequate staffing, and insufficient access to specialist support—is essential to enable teachers to deliver high-quality education. Additionally, school leadership should actively support and empower teachers, creating the conditions necessary for equitable education to emerge and be sustained.

## Conclusion

This review underscores the central role of teacher self-efficacy in facilitating equitable and inclusive education for autistic students. Consistent with the OECD's (2018) definition, equity is understood not as a fixed placement model, but as a responsive and flexible approach that supports students’ participation, development, and well-being in the most appropriate context. Inclusive education, from this perspective, encompasses both rights-based and needs-based dimensions. The reviewed studies suggest that, although teachers express a willingness to support autistic students, their self-efficacy is often undermined by limited autism-specific training, systemic constraints, and insufficient support. Strengthening teacher self-efficacy through sustained professional development and institutional support emerges across the literature as a key strategy for improving instructional practice. Enhanced self-efficacy may also contribute to better student well-being and reduce the risk of exclusion, including school avoidance and absenteeism—outcomes linked to negative school experiences and inadequate teacher understanding (Atkinson et al., 2024; Brede et al., 2017; Gray et al., 2023; Holmes, 2022; Lynam et al., 2024).

## Supplemental Material

sj-docx-1-dli-10.1177_23969415251392318 - Supplemental material for A Systematic Research Review on Teachers’ Self-Efficacy in Educating Autistic Students Supplemental material, sj-docx-1-dli-10.1177_23969415251392318 for A Systematic Research Review on Teachers’ Self-Efficacy in Educating Autistic Students by Linda Petersson-Bloom and Erika Hansson in Autism & Developmental Language Impairments

sj-docx-2-dli-10.1177_23969415251392318 - Supplemental material for A Systematic Research Review on Teachers’ Self-Efficacy in Educating Autistic Students Supplemental material, sj-docx-2-dli-10.1177_23969415251392318 for A Systematic Research Review on Teachers’ Self-Efficacy in Educating Autistic Students by Linda Petersson-Bloom and Erika Hansson in Autism & Developmental Language Impairments

sj-docx-3-dli-10.1177_23969415251392318 - Supplemental material for A Systematic Research Review on Teachers’ Self-Efficacy in Educating Autistic Students Supplemental material, sj-docx-3-dli-10.1177_23969415251392318 for A Systematic Research Review on Teachers’ Self-Efficacy in Educating Autistic Students by Linda Petersson-Bloom and Erika Hansson in Autism & Developmental Language Impairments
