# Supplementary material for: A Systematic Research Review on Teachers’ Self-Efficacy in Educating Autistic Students
Source: Autism Dev Lang Impair. 2025 Oct 29;10:23969415251392318. doi: 10.1177/23969415251392318 (PMC12576102; doi:10.1177/23969415251392318)
Supplement: sj-docx-1-dli-10.1177_23969415251392318 - Supplemental material for A Systematic Research Review on Teachers’ Self-Efficacy in Educating Autistic Students [file sj-docx-1-dli-10.1177_23969415251392318.docx]

Supplementary material: Data extraction matrix included studies

| # | Author/  year | Country | Placement^a^ | | | Participants | School year^b^ | | Methods for data collection/ type of scale | Research design^c^ | | | | Research focus/aim | Findings/ Conclusion | Comment | Quality appraisal | | |
| --- | --- | --- | --- | --- | --- | --- | --- | --- | --- | --- | --- | --- | --- | --- | --- | --- | --- | --- | --- |
|  |  |  | **^1^** | **^2^** | **^3^** |  | 1 | 2 |  | 1 | 2 | 3 | 4 |  |  |  | 1 | 2 | |
| 1 | Accardo et al.  (2017) | USA | x | x | x | 112 teachers | x | x | Questionnaires: e Reading Teaching Efficacy Instrument (RTEI) and the Effective Practices Survey | x |  |  |  | identify predictors of (1) teacher perceived self-efficacy in teaching reading and (2) teacher perceived outcome expectancy regarding their professional ability to improve the reading comprehension | only 5% of teachers reported a high level of self-efficacy in teaching reading comprehension. Teacher preparedness to use effective practices, years of experience and administrator support emerged as significant predictors of self-efficacy (R² = .43. Importance of providing ongoing support to teachers of learners with ASD, including support from school principals and administrators, and ongoing professional development in using effective practices to teach reading comprehension to learners with ASD |  | x | |  |
| 2 | Alallawi et al.  (2022) | UK |  |  | x | 10 special educators, 5 teachers and 5 teaching assistants. 6 females in total (3 teachers) | x | x | Semi structured interviews |  | x |  |  | explore the experiences of special educators who had implemented the TEN-DD intervention with their autistic students | Educators reported an increased sense of competence in their teaching skills, evident in greater satisfaction and increased self-efficacy. Furthermore, there was a strong interest in continuing to use the numeracy intervention with students. There were also implementation challenges with TEN-DD, including students’ challenging behavior. | Teaching Early Numeracy to children with Developmental Disabilities (TEN-DD) programme | x | |  |
| 3 | Alhumaid (2021) | Saudi Arabia |  |  |  | 214 PE teachers, 188 male teachers and 26 female teachers. In different regions of SA | x | x | Questionnaire: Arabic version of the Physical Educators’ Self-Efficacy Towards Including Students with Disabilities-Autism (PESEISD-A) instrument | x |  |  |  | Determine the levels of SE toward including students with autism among Saudi PE teachers and (2) to identify the predictors of Saudi PE teachers’ SE toward including students with autism in PE classes. | moderate level of SE among the participants toward including students with autism in PE classes. Significant gender differences were evident: male teachers expressed higher levels of SE toward including students with autism than female teachers. PE teachers who have a good level of academic preparation are more likely to report higher levels of SE toward the inclusion of students with autism |  | x | |  |
| 4 | Alkeraida (2023) | Saudi Arabia | x |  |  | 4 male teachers at two primary schools | x |  | General interview, pre- and post-lesson interview, observation & Teacher Efficacy for Inclusive Practice (TEIP). |  |  |  | x | illustrate and analyse teachers’ decision-making in relation to the participation of students with autism across several relevant cases at the classroom level. | holding a negative attitude and a lower level of teaching efficacy was found to be an obstacle to the in-class participation of students with autism. Teachers with negative attitudes and a lower level of teaching efficacy made little to no effort to promote the participation of students with autism, thereby increasing their risk of being excluded. | a longitudinal multiple case studies design over the academic year of 2017/18. Triangulation.  MMAT assed as qualitative. | x | |  |
| 5 | Anglim et al. (2017) | Ireland | x |  |  | 6 female teachers | x |  | one-to-one, open-ended, semi-structured interviews. |  | x |  |  | elicit the lived experiences of Irish primary school teachers in relation to teaching children with ASD in mainstream education + highlight any perceived barriers to inclusion and to identify supports that teacher revealed to be useful in supporting inclusive practices in their schools. | two-thirds of the primary school teachers (4) interviewed were apprehensive and lacked confidence at the initial prospect of teaching a child with ASD. Teachers believe that they needed to feel informed and prepared and experienced pressure and flet uncertain in managing the behaviour of autistic students. All described growing in confidence from the experience of teaching a child with ASD |  | x | |  |
| 6 | Baek et al. (2024) | USA | x | x | x | 180 teachers (male=58, female=121, other=1). Teachers were employed in North America=34, South America=1, Europe= 134, Asia=4 & Australia | x | x | Questionnaires.  Autism Self-Efficacy Scale for Teachers (Ruble et al.2013), Willingness to Accommodate scale developed by Lombardi and Murray (2011), teachers’ general empathy adopted the scale by Archer and Finger (2018) | x |  |  |  | Examines teachers’ willingness to provide accommodations to autistic students and their self-efficacy to teach these students. + examines which factors are associated with teachers’ willingness to provide accommodations and self-efficacy. + test an online intervention. | teachers expressed a positive attitude towards providing accommodations for students with autism across diverse teaching settings and regions of employment. However, they lacked confidence in teaching these students, and those with direct experience had greater self-efficacy. The results indicated that a short online intervention, can effectively increase teachers’ self-efficacy. The support teachers receive from their schools is important in improving their attitudes towards students with disabilities. | Teachers from different countries participated. All grades (from preschool to high school. Majority elementary to high school. | x | |  |
| 7 | Bertuccio et al. (2019) | USA, | x |  |  | Teachers (59) and paraprofessionals (28)N=87, from a school district | x | x | Questionnaires: an autism knowledge questionnaire +the Teacher Self-Efficacy for Autism Scale (TSEAS; Love, 2016) + a Satisfaction questionnaire (after training) | x |  |  |  | examine a priori differences in knowledge and self-efficacy for teachers and paraeducators + evaluate changes in knowledge and self-efficacy across time for each of these groups following trainings, + compare perceived professional development needs for teachers and paraeducators. | Results for teachers and paraeducators followed similar patterns in that knowledge of autism increased significantly but did not sustain over time. Levels of self-efficacy increased significantly following the practical training. Teachers demonstrated higher levels of knowledge compared to paraeducators across time, while levels of self-efficacy converged. |  | x | |  |
| 8 | Bitska et al. (2017) | Australia | x |  |  | 23 educators (52% classroom educators, special education staff 30%, heads of special education 13% & deputy principals 5 %). 87% female. | x | x | Questionnaire: Measure of FBA knowledge (developed by first author) + Klassen and Ming Chui’s (2010) 20-item Teacher Sense of Efficacy Scale (TSES) + Self-confidence measure (designed for the study) | x |  |  |  | efficacy of delivering autism-focused Functional Behaviour Assessment (FBA) training within a six-session professional development (PD) framework was investigated | Significant relationships were detected between pre-training measures of educator SE and SC (r (21) = .61, p = .002), and post-training measures of SE and SC (r (21) = .52, p = .01). FBA training had a positive impact on educators’ self-efficacy and confidence, with significant improvements observed in a short time. |  | x | |  |
| 9 | Bond et al. (2017) | UK | x | x |  | 30 educators completed questionnaire (teachers N=15, teachers assistant N=15, majority 86% female) + took part in the training, 20 educators also participated in semi-structured interviews. | x | x | Questionnaire: developed and piloted a questionnaire for use in the project. Based on the teacher self-efficacy section in the Instruction of Students with Autism Scale (Caywood, unpublished).  Semi-structured interviews at 3 points. |  |  |  | x | Explores how training content, educators’ characteristics & organisational factors interact in the professional development of specialist school staff working with students with ASD. | The quantitative data demonstrates how the relatively highly skilled participants who initially had positive perceptions about working with pupils with ASD were able to extend their skills and sense of self-efficacy. The findings showed increased perceived self-efficacy for the sample as a whole and particularly among the TAs. Supports training focusing on development of skills, knowledge and efficacy, complemented by environments which enable training to be applied and embedded. | MMAT assessed as quantitative descriptive. | x | |  |
| 10 | Boujut et al. (2017) | France | x | x | x | 203 teachers in metropolitan France 62%, Guyana 16%, Martinique 16%, New Caledonia 6% | x | x | Questionnaires: self-efficacy was measured with a scale (GSE) developed by Walliser, Schwarzer, and Jerusalem (1993), stress was measured with the Appraisal of Life Events Scale (ALES) created by Ferguson et al. (1999) +the Ways of Coping Checklist-Revised, created by Vitaliano et al. (1985) + The Maslach Burnout Inventory (MBI, Maslach & Jackson,1981) | x |  |  |  | test the mediating role played by perceived stress and coping strategies in the relationship between perceived self-efficacy and burnout. How teaching children with ASD can affect teachers’ burnout levels | When teachers have a low level of perceived self-efficacy, they tend to implement more emotion-focused coping strategies, which leads to more burnout. The lower the teachers’ level of perceived self-efficacy, the more a stressful situation is perceived as a threat or loss, which causes more emotional exhaustion. Results suggest that the perception teachers have about school inclusion and their coping strategies (resulting in behavioural, cognitive, and emotional reactions) are directly involved in their adjustment in terms of professional exhaustion. Moreover, these factors reinforce the positive effects of perceived self-efficacy on teachers’ mental health. |  | x | |  |
| 11 | Breeman et al. (2016) | The Netherlands |  |  | x | 58 teachers, 72% females | x |  | Questionnaire: Student Engagement and Classroom Management sub-scales of the short version of the Teachers’ Sense of Efficacy Scale (TSES; Tschannen-Moran & Hoy, 2001), Maslach Burnout Inventory, the Utrecht’s Burn-Out Schaal for teachers (UBOS-L; Schaufeli & Van Dierendonck, 2000) + Student–Teacher Relationship Scale (STRS; Koomen, Verschueren, & Pianta, 2007). | x |  |  |  | examine the impact of the Good Behaviour Game on primarily children with ADHD and ASD, and their teachers | Teachers’ outcomes, we found a significant effect of the GBG on teachers’ self-efficacy in student engagement. No impact of the GBG was found on teachers’ self-efficacy in classroom management, emotional exhaustion, personal competence, or teacher– child closeness. | Focus on teacher data | x | |  |
| 12 | Cappe et al. (2017) | Canada | x | x | x | 115 teachers, 93.9% females. | x | x | Questionnaires: the General Self-Efficacy Scale (GSES; Jerusalem & Schwarzer, 1992; Schwarzer & Jerusalem, 1995)  Empathy Quotient (EQ), Appraisal of Life Events Scale (ALES), Questionnaire de soutien social perçu, QSSP; (Cappe et al., 2011; Koleck, 2000), Ways of Coping Checklist - Revised (WCC-R), Maslach Burnout Inventory was used to assess teacher burnout | x |  |  |  | Deepen the understanding of burnout in French-Canadian teachers of students with ASD. Comparing teachers’ dispositional factors (e.g., self-efficacy, empathy) and transactional factors (e.g., perceived stress, social support, coping strategies), alongside burnout +how these factors are related to and predict various dimensions of burnout. | Teachers of students with ASD did not exhibit more signs of burnout than regular teachers. Perceived stress and social support predict burnout among teachers of children with ASD in mainstream classes while self-efficacy also predicts burnout among teachers in specialized settings. | Also includes preschool. | x | |  |
| 13 | Cappe et al. (2021) | France & Canada | x | x | x | 172 teachers, 92.4% females. France (n=86, Quebec (n=86) |  |  | Questionnaires: the General Self-Efficacy (GSE) scale, Appraisal of Life Events Scale (ALES) (Ferguson, Matthews, and Cox, 1999), Perceived Social Support Questionnaire (PSSQ; Koleck, 2000), Ways of Coping Checklist - Revised (WCC-R), Maslach Burnout Inventory | x |  |  |  | highlight differences but also similarities between the adjustment of teachers in France and in Quebec working in different educational and cultural systems. Focusing on assessing stress, social support, coping, and burnout. | self-efficacy was lower for teachers in France than for teachers in Quebec, but only in the group of teachers who taught a regular class including an autistic student. Adjustment among Quebec teachers was better, highlighting the need to consider educational systems and cultural context when examining teacher well-being in inclusive education. |  | x | |  |
| 14 | Catalano et al. (2023) | USA | x |  |  | 289 teachers (in-service, N=156 & pre-service, N=133. In total 96.5% females. | x |  | Questionnaire: TSE-ASDI/EC & Teachers’ sense of efficacy scale (TSES) | x | x |  |  | develop and validate the Teacher Self-efficacy for Teaching Students with ASD in Inclusive Early Childhood Classrooms Scale: TSE-ASDI/EC which measures pre-service and in-service general education teachers’ self-efficacy for teaching students diagnosed with ASD. | Both pre-service and in-service teachers with special education preparation had statistically significantly higher mean scores on the TSE-ASDI/EC Scale than their counterparts without this preparation+ participants with experience with students with ASD had statistically significantly higher mean scores on the TSE-ASDI/EC Scale than teachers without this experience.  This 16-item TSE-ASDI/EC Scale was highly reliable. | Focusing on the results from questionnaire. Study also includes cognitive interviews as part of the development. MMAT assessed as quantitative descriptive. | x | |  |
| 15 | Cook & Ogden (2021) | UK | x |  | x | 12 teachers (6 general & 6 special school). 4 females. |  |  | Semi-structured interviews |  | x |  |  | experiences of teachers from contrasting school settings regarding autistic students, and in particular their self-efficacy in relation to teaching students on the autistic spectrum | whilst inclusive educational ideologies, sufficient staff expertise and training and conducive physical environments enabled the implementation of strategies to facilitate achievement, a one-size-fits-all approach, workload pressures and heterogeneity of ASD characteristics tended to affect their self-efficacy and hence their ability to meet the needs of the students. |  | x | |  |
| 16 | Corona et al. (2017) | USA |  |  |  | 80 school professionals (special education teachers, n=14, general teachers, n=12, subject teachers, n=5) other professionals e.g, psychologist n=12, Speech language pathologist, n=10, administrators, n= 8. |  |  | Questionnaire: Autism Self-Efficacy Scale for Teachers (ASSET) & Autism Knowledge Questionnaire | x |  |  |  | Predictors of school professionals’ self-efficacy for working with students with ASD were examined + the impact of training on ASD and Prevent–Teach–Reinforce (PTR) on school professionals’ knowledge about ASD and ASD-related self-efficacy was assessed. | Prior training in ASD and Positive Behavior Support (PBS) emerging as significant predictors of self-efficacy for professionals. + school professionals’ knowledge and self-efficacy increased following participation in a training on ASD and PBS, providing preliminary evidence that training in EBPs may enhance school professionals’ belief in their abilities to successfully work with this group of students. | Not stated grade or placement. Also, findings include all the participants. | x | |  |
| 17 | Devi & Ganguly (2024) | Australia |  |  |  | 16 teachers (8 preservice teachers & 8 recent graduated teachers) |  |  | Interviews |  | x |  |  | Gain insights on experiences and perceptions of inclusion of students with ASD. | Factors that affect SE: Teacher education programmes that incorporate more hands-on experiences for novice teachers, oﬀering a compulsory course on teaching students with ASD and teacher placements where adequate and eﬀective mentorship provided + prior contact with individuals with ASD, e.g. being a parent with a child with ASD, or has worked as a teacher aide or as a volunteer worker for students with ASD |  | x | |  |
| 18 | Dille (2013) | USA | x | x | x | 48 teachers/ 16 in each group (including control group) |  |  | Questionnaires (4) developed by the researcher, e.g., Teacher Knowledge of Autism and the Educational Treatment of Autism Questionnaire, Teacher Self-Efficacy of Use of Instructional Methods, Strategies, and Supports for Students with Autism | x |  |  |  | compare the effectiveness of two curricular models of professional development to increase teacher repertoires for instructing students with autism. Blended & Bahavioral models were compared. regarding increasing teacher knowledge, teacher self-efficacy, teacher self-report and teacher application of instructional methods, strategies, and learning supports. | Both the Blended and Behavioral groups showed significantly greater mean increases compared to the Control group in teacher knowledge, teacher self-efficacy, and self-reported use of instructional methods. Although differences between Blended and Behavioral groups weren't significant, the Blended group generally had higher mean increases. |  | x | |  |
| 19 | Egan & Kenny (2022) | Ireland |  | x |  | 10 participants, 6 teachers and 4 principals | x |  | semi-structured interviews |  | x |  |  | examine the experiences and perspectives of principals and teachers working in special classes for autistic pupils across a range of Irish primary schools | Study identified factors impacting teacher wellbeing highlighting that teachers often experience isolation, stress, and inadequate preparation due to limited autism-specific training and inconsistent school support. Recommendations include enhancing continuous professional development, cultivating collegial and inclusive school cultures, and providing practical and emotional support to improve teacher self-efficacy and educational outcomes for autistic students. | Bothe teachers and principals represented in the findings. | x | |  |
| 20 | Emmons & Zager (2018) | USA |  |  |  | 104 special education teachers, related service providers, and administrators. | x | x | Questionnaire: The Self-Efficacy Study Scale was developed | x |  |  |  | perceived self-efficacy concerning collaborative expertise in delivering educational programming for students with autism, pre and post participants’ engagement in the Autism Specialist Program | The program significantly increased participants self-efficacy in creating program change and collaborating with general educators, with smaller improvements in traditional areas like classroom behavior management and collaboration with other special educators, indicating the training strongly enhanced participants' collaboration self-efficacy. | Participants were employed in urban high-need schools, working with children and youth in Grades K-12 | x | |  |
| 21 | Hinton et al. (2008) | Australia |  |  |  | 58 teachers (45 female & 13 male). 25 females and 4 males in intervention group and 20 females and 9 males in the wait-list control group | x | x | Questionnaire: a teacher-report measure designed for the study | x |  |  |  | The teacher training intervention aimed to increase teacher competence in managing the problem behaviours associated with Asperger’s syndrome, as manifested in a classroom setting. Variables of interest were number of problem behaviours, success of teacher strategies used to manage problem behaviours and teacher self-efficacy in managing behaviours | The control group’s confidence remained unchanged, while the intervention group showed a significant increase from pre to six-weeks post workshop. Concerning levels of confidence in their ability to manage the student in the classroom. | SE is the outcome | x | |  |
| 22 | Horan & Merrigan (2019) | Ireland |  | x |  | 50 teachers responded to questionnaire, 7 teacher interviews (7 female & 1 male). | x |  | Questionnaire: Teacher Efficacy for Inclusive Practices (TEIP) + semi-structured interviews |  |  | x |  | impact of professional development on teachers perceived levels of efficacy to teach inclusively in a special class setting for students with ASD | variety of reported teacher efficacy levels in both the qualitative and quantitative data. highly trained’ teachers had significantly higher perceived efficacy levels compared to those who had received ‘little or no training + teacher efficacy is enhanced through professional development + perceptions of teacher efficacy levels were heavily dependent on getting to know the child as an individual | MM: qual + quant =compared, merged and analysed simultaneously.  inferential statistical analyses based on 39 responses (teachers that disclosed their training details) | x | |  |
| 23 | Humphrey & Symes (2013) | UK | x |  |  | 53 participants (21 male & 32 female). 32 teachers, 11 management & 19 SENCOS |  | x | 58 item questionnaires, adapted from McGregor and Campbell’s (2001) study. | x |  |  |  | Examine the experience, attitudes and knowledge of school staff in relation to inclusive education for pupils with autistic spectrum disorders (ASDs) in mainstream secondary schools | more positive responses than have been reported in previous studies (concerning inclusion). SMs and SENCOs reported greater self-efficacy in teaching pupils with ASD and in coping with behaviours associated with ASD |  | x | |  |
| 24 | Johnson et al. (2021) | USA | x |  |  | 56 teachers completed both pre-and post-test. | x |  | Questionnaire: Teacher self-efficacy was measured by adapting a survey from a study regarding teacher self-efficacy in teaching nutrition (Brenowitz & Tuttle, 2003) | x |  |  |  | Determine the effect of teacher professional development for working with students with autism spectrum disorders and teacher self-efficacy in the general education classroom. | Training significantly improved teacher self-efficacy for working with students with autism in inclusive classrooms, highlighting the need for expanded professional development for general education teachers. | Teacher ranging from pre-kindergarten. | x | |  |
| 25 | Kingsdorf et al. (2024) | Europe first author Czech Republic |  |  |  | 209 physical educators |  |  | Questionnaire: self-assessed knowledge and attitudes survey.  Based on PESEISD-A | x |  |  |  | examine self-assessed knowledge and attitudes of PE teachers concerning autistic students. | responses showed moderate levels of self-efficacy. novel look at challenges and practices verified that respondents do not consider themselves skilled in implementing strategies for supporting autistic children in inclusive physical education classrooms |  |  | |  |
| 26 | Kisbu-Sakarya &Doenyas (2021). | Turkey | x |  |  | 763 teachers (67–6 % female) |  |  | Questionnaires:  Autism Self-Efficacy Scale for Teachers (ASSET) was used (Ruble et al., 2013) + Autism Attitude Scale for Teachers (AAST) (Olley et al., 1981) + Two questions that were adapted from Jones (2009) assessed the behavioral intention. | x |  |  |  | The study examined how teacher training in special education strategies for ASD influences mainstream teachers’ intentions toward inclusive education, focusing on the mediating roles of attitudes and autism self-efficacy. | teacher special education training programs can be effective in increasing teachers’ behavioral intentions toward inclusive education through increasing their self-efficacy for teaching students with ASD |  | x | |  |
| 27 | Latorre-Cosculluela,et al. (2022) | Spain | x |  |  | 454 teachers | x |  | See below! | x |  |  |  | “The aim of this study was to explore the relationships between the perceived efficacy of a set of practices specifically aimed at children with ASD and the perceived drivers and attitudes toward their full inclusion.” | “The results show that greater efficacy of the practices implemented with children with ASD results in more positive attitudes toward the education of these children in inclusive settings. Similarly, drivers of inclusion also improve teachers’ attitudes toward these children.” | Note that the second study by Latorre et al. (2023) has the same sample (28) | x | |  |
| 28 | Latorre-Cosculluet al. (2023) | Spain | x |  |  | 454 teachers | x | x | Online questionnaire  Scale: 0–10 Likert-type scales for:  Perceived efficacy of inclusive practices  Intensity of use  Perceived achievement of inclusion | x |  |  |  | “The objective of this study is to analyse the possible relationships between the perceived efficacy by teachers about a set of inclusive practices for students with ASD, the use of these practices today and, finally, the perceived achievement of educational inclusion.” | “We have noted a positive relationship between mainstream classroom teachers’ trust of their inclusive practices and achieving quality educational inclusion of children with ASD.”  “This intensity of use in the classroom also means that teachers perceive the achievement of inclusion for students with ASD.” |  | x | |  |
| 29 | Lisak Šegota, et al., (2022) | Croatia, North Macedonia, Poland | x |  | x | 242 experienced primary school teachers | x |  | The instrument was informed by a literature review on autism teacher training (referencing Lessner Lištiaková & Preece, 2019) | x |  |  |  | “A survey of experienced teachers working directly with children with autism in both inclusive and special schools was undertaken… Teachers identified a need for appropriate teacher education regarding theory, relevant practical strategies and mentorship/supervision.” | “Many experienced teachers supporting children with autism lack such education and... feel unconfident in meeting the needs of these children. There is an urgent need to provide teachers with appropriate education opportunities at both ITE and CPD levels and to equip them with effective practical strategies based on sound evidence-based principles of good autism practice.” |  | x | |  |
| 30 | Love et al (2020) | USA |  | x |  | 44 special education teachers | x |  | Self-report and observational tools.  ASSET (Autism Self-Efficacy Scale for Teachers) –  TERS (Teacher Engagement Rating Scale) – observer-rated,  ITS (Index of Teaching Stress)  PET-GAS (Goal Attainment Scaling) – observational | x |  |  |  | “Our primary research question asked about the relationships between self-efficacy for teaching students with ASD and teacher stress, teacher engagement, and student IEP outcomes.” | “Teachers who believed they could teach students with ASD also were more likely to engage positively with their students with ASD and those students achieved higher student IEP goal attainment outcomes.” |  | x | |  |
| 31 | Love, (2019) | USA & Australia | x  USA |  | x AUS | U.S. sample (N = 120): General and special education teachers, ages 5–18; mostly female (81%); 38% elementary, 20% high school, 12% special education.  Australian sample (N = 85): Special educators, 94.1% female; variety of teaching roles including elementary, high school, and special education​. | x | x | Online surveys  TSEAS (new scale for ASD self-efficacy) – 12-item, 4-point Likert (validated: ω = .91–.96)  TSES (general self-efficacy)  Job Satisfaction Index  Self-Regulation Scale | x |  |  |  | "The purpose of this investigation was to develop an instrument that can [be] used to measure teachers’ self-efficacy for effectively working with students with ASD | "This conclusion demonstrates the need for a researcher to use a student-specific scale and points to the importance of a teacher self-efficacy scale that is specific to teaching students with autism." |  | x | |  |
| 32 | Lu et al (2020) | China | x |  |  | 386 primary school teachers | x |  | Standardized self-report questionnaires:  Autism Stigma and Knowledge Questionnaire (ASK-Q) — dichotomous (true/false)  Autism Attitudes Scale for Teachers (AAST) — 5-point Likert scale  Professional self-efficacy scale — 5-point Likert scale | x |  |  |  | "This study made the following assumptions: (i) knowledge, attitude and professional self-efficacy are significantly correlated, (ii) attitude mediates the relationship between knowledge and professional self-efficacy and (iii) attitude moderates the relationship between knowledge and professional self-efficacy." | "This study found that knowledge of ASD and attitude toward children with ASD had significant predictive effects on professional self-efficacy. Attitude both mediated and moderated the influence of knowledge on professional self-efficacy." |  | x | |  |
| 33 | Maddox, & Marvin (2013) | USA | x |  |  | 28 trainees: Special education teachers (n=18), speech-language pathologists (n=4), occupational therapists (n=3), school psychologist (n=1), inclusion facilitator (n=1), and special education consultant (n=1).  21 mentors: Including teachers, coordinators, speech therapists, autism specialists, etc. | x |  | Pre/post surveys, observation checklists, mentor reports, portfolios.  The study used the "Effective Practices Checklist for Students with Autism", which was developed by the ASDN state coordinator based on recommended practices from credible sources like the National Research Council (2001) and Iovannone et al. (2003) |  |  |  | x | "The primary goal of this evaluation study was to examine the effects of the ASDN’s STEPS training as documented by the program’s trainees themselves and their program mentors." | "STEPS training significantly improved Trainees’ perceived knowledge and skill, as well as some Trainees’ classroom practices. | MMAT assed as quantitative descriptive. | x | |  |
| 34 | Nemček, et al (2024) | Slovakia | x |  |  | 117 Physical educators |  | x | PESEISD-A questionnaire (Taliaferro et al., 2010), validated for Slovak context | x |  |  |  | “The study’s objective was to determine the level of self-efficacy toward the inclusion of students with autism spectrum disorders among physical education teachers in Slovakia and to compare it between teachers with and without experience.” | “The present research indicates that regardless of PE teachers’ experience, their self-efficacy toward including students with ASD in PE classes does not significantly differ.”  “Both groups of teachers perceive themselves as moderately capable of including students with autism in their PE classes...” |  | x | |  |
| 35 | Nolan, & Hannah, (2019) | Scotland | x |  |  | 35 participants  Nursery sector: 17 participants  Primary sector: 5 participants  Secondary sector: 13 participants  Roles included: teachers, early years educators, pupil support assistants, school leadership (e.g., headteachers) | x | x | SACIE-R scale: 15 items, 4-point Likert scale  TEIP scale: 18 items, 6-point Likert scale  Post-only questionnaire:  Open-ended and 1–10 scale questions on confidence, strategy use, and training usefulness |  |  | x |  | “The study aimed to assess the impact of a training programme on:  (1) school staff’s attitudes, sentiments and concerns…  (2) school staff’s efficacy in supporting pupils with Autism…  (3) school staff’s perceptions of the usefulness of the training, application of knowledge, and effectiveness of strategies.” | “Across the overall sample there was a significant improvement in participants’ efficacy... post-training.” (p. 101)  “Training has had a positive impact on participants’ espoused practice...” |  | x | |  |
| 36 | Oh, & Kozub,  (2010) | South Korea and USA | x |  |  | 368 physical educators  Korea: 229 participants (mostly male, secondary level)  U.S.: 139 participants (mostly female, elementary level)  Roles included general PE teachers, adapted PE teachers, and dual health/PE teachers | x | x | Self-report questionnaire (the Difficult Behavior Self-Efficacy Scale)  To standardize context, participants watched a video vignette depicting a student with autism spectrum disorder (ASD) displaying aggressive behavior (e.g., throwing a basketball at a teacher), to focus responses on teacher efficacy in an autism-specific situation | x |  |  |  | “This study was designed to examine the psychometric properties of this scale with a diverse sample of physical educators. A second purpose was to study the factor structure for both the Korean and United States (U.S.) versions of the scale…” | “The one-factor model for the Difficult Behavior Self-Efficacy Scale… has factorial validity and reliability when used to study these physical educators from Korea and the U.S.”  “Metric invariance was also confirmed… however, the items lacked scalar invariance across groups.” |  | x | |  |
| 37 | Park, et al (2019) | South Korea |  |  | x | Students: Ranged from 136 to 250 per school; all had developmental disabilities  Teachers: 42–70 per school; special educators  PBS mentors: University students majoring in special education  PBS leadership teams: Included school administrators, teachers, and consultants | x | x | Methods:  Pre/post video observation (academic engagement)  Goal Attainment Scaling (GAS)  Fidelity checklists  Qualitative data (e.g., meeting notes, interviews)  Likert scales for teacher efficacy and classroom management (4- or 5-point)  GAS (5-point individualized performance scale)  Benchmark of Quality (BoQ) for fidelity (51 items scored 0–2) |  |  | x |  | “The purpose of this study was to present processes and outcomes of School-Wide Positive Behaviour Support across multiple years.” | “The Special School-Wide Positive Behaviour Support project resulted in improved academic engagement behaviors and satisfactory goal attainment in the students as well as enhanced efficacy and class management skills in the teachers.” |  | x | |  |
| 38 | Parsons, et al. (2016) | USA | x |  |  | 94 general education teachers (one incomplete response excluded) | x | x | Online email-distributed survey  Teacher Efficacy for Inclusive Practice (TEIP) Scale | x |  |  |  | “The purpose of this study was to determine if there was a relationship between the amount of special education training obtained by general educators and their efficacy levels for classroom management and inclusive instructional strategy use with their included students with autism.” | “Data from this study demonstrated a strong positive correlation between general education teacher classroom management efficacy and inclusion instructional strategy use for students with autism.”  “Teachers who have had one university course in special education demonstrated the highest levels of efficacy. […] Educators with the highest level of training, special education teacher certification, reported a significant drop in efficacy for both dependent variables. |  | x | |  |
| 39 | Rakap, et al (2018) | Turkey | x |  |  | Total participants: 478 general education teachers  School levels: 92 preschool, 105 primary, 126 middle, 155 high school teachers  Gender: 58% female, 42% male | x | x | Online survey with Likert scales. Non-validated; Demographics  Knowledge about causes and diagnosis of ASD  General knowledge and perceptions of ASD  Knowledge of evidence-based practices and training needs | x |  |  |  | “This study was designed to examine Turkish teachers’ general knowledge and perceptions about autism spectrum disorder, explore their knowledge about evidence-based practices in ASD, and examine their training needs to serve children with ASD. | “Findings of the current study showed that Turkish general education teachers who participated in the current study are not appropriately trained to work with children with ASD. The lack of adequate training in ASD warrants the development of professional development or certification programs…” |  | x | |  |
| 40 | Rakap, et al. (2015) | USA | x |  |  | 33 in-service teachers (mostly female, Caucasian)  Teaching in special education or related roles  All had bachelor’s degrees or higher and worked with students with ASD | x |  | Self-assessment surveys (pre-post) → 5-point Likert scale  Use of Knowledge in Practice Survey → 4-point Likert scale  Satisfaction Surveys (3 times) → 5-point Likert scale  No formal external validation studies reported for most instruments. | x |  |  |  | “The purpose of this article is to describe the development, implementation, and second-year evaluation of a web-based in-service PD program (Project ACE) designed to train teachers currently working in the field to meet the unique and diverse needs of children with ASDs. | “Preliminary findings... showed that the web-based PD program... was effective in helping teachers develop and improve their competencies, knowledge, and skills. Moreover, teachers felt comfortable using the teaching strategies they learned... and reported some application of these strategies in their practices.” |  | x | |  |
| 41 | Rodden, et al. (2019) | Ireland | x |  |  | 10 mainstream secondary school teachers |  | x | Semi-structured interviews  No numerical scale – data are purely qualitative, using discourse analysis |  | x |  |  | “This study is an exploratory study that endeavoured to examine both the theoretical and practical knowledge base of teachers who educate students with ASD at post-primary level.” | “Mainstream teachers do not feel sufficiently supported in their training and also in their teaching practice to provide an inclusive education for students with SEN, such as ASD.” |  | x | |  |
| 42 | Ruble, et al. (2013) | USA |  | x |  | N = 44 special education teachers | x * |  | ASSET (Autism Self-Efficacy Scale for Teachers): 30 items, originally rated on a 0–100 scale; also recoded into a 6-point Likert scale  ITS (Index of Teaching Stress) and MBI (Maslach Burnout Inventory) used 5-point and 7-point Likert scales, respectively  Data collected via self-report questionnaires | x |  |  |  | “The purpose of the current study was to evaluate a new measure, the Autism Self-Efficacy Scale for Teachers (ASSET) for its dimensionality, internal consistency, and construct validity...” | “The ASSET is a promising tool... Preliminary support for the reliability and one-dimensionality of scores generated by the ASSET was found. Correlations with stress (ITS) were significant and in the expected direction, but correlations with general burnout (MBI) were weak or absent.” | *Primary school  ages 3-9 | x | |  |
| 43 | Ruble, et al (2011) | USA |  | x |  | 35 special education teachers | x |  | Self-report questionnaires using established instruments:  Teacher Interpersonal Self-Efficacy Scale (TISES) – 6-point Likert scale  Maslach Burnout Inventory (MBI) – 7-point Likert scale  Multifactor Leadership Questionnaire (MLQ) – 5-point scale  Objective data (e.g., years of experience) also collected via background form | x |  |  |  | “The purpose of the current study is to explore the relationship between three factors hypothesized to be related to self-efficacy and the efficacy beliefs reported by teachers of students with autism.” | “Significant associations were observed between physiological/affective states and self-efficacy, but no associations were observed for the other sources.” | * Primary school (age 3-9 | x | |  |
| 44 | Ryan & Mathews(2022a) | Ireland |  |  |  |  |  |  |  | x |  |  |  | “What components of educating learners with A/ASD are special class teachers most and least confident in? | “Participants felt most confident in their ability to use visual supports to foster student independence, to describe their student’s characteristics that relate to A/ASD, and to communicate and work effectively with parents or caregivers. Conversely, they felt least confident in their abilities to train peer models, to teach play skills, and to translate assessment information into teaching objectives to the students. | The same sample as in Ryan & Mathews(2022b) 45. |  | |  |
| 45 | Ryan & Mathews(2022b). | Ireland |  | x |  | 139 ASD class teachers. | x |  | Online questionnaire, combining:  Autism Self-Efficacy Scale for Teachers (ASSET) – 30 items, 6-point Likert scale  Principal’s Instructional Support Scale – 3 items, 5-point Likert scale  Multiple-choice and checkbox items for teaching experience and CPD engagement | x |  |  |  | “This study measured perceptions of TSE among ASD class teachers in Ireland and investigated how TSE is affected by three independent variables... special class teaching experience, engagement with CPD pertaining to autism, and perceptions of principals’ instructional support.” | “The present study identified significant positive correlations between the independent variables of years’ special class teaching experience, engagement with CPD pertaining to autism, and perceptions of instructional support, with the dependent variable of TSE. | CPD stands for continuing professional development | x | |  |
| 46 | Selvaganapathi, et al (2019 | Malaysia | x |  |  | 451 teachers, 73.2% female. | x |  | The questionnaire was self-developed, not based on any named or published psychometric instruments.  1. Knowledge of Normal Child Development  2. Knowledge of Autism Spectrum Disorder (ASD)  3. Attitudes Toward Children with ASD  4. Interest and Perceived Self-Efficacy  5. Awareness of Organizations  6. Awareness of Interventional Approaches | x |  |  |  | “This study aims to assess teacher’s knowledge and perception towards children with autism spectrum disorder (ASD) in Malaysia.” | “We found that teachers did, in fact, recognize their own lack of knowledge of ASD and this awareness co-existed with an interest in increasing their knowledge and skills in this area.” | Yes |  | |  |
| 47 | Siu, & Ho, (2010) | Hong Kong |  |  | x | 115 teachers, 95% female | x | x* | ATPQ – Autism Treatment Philosophy Questionnaire. 6-point Likert scale.  Teacher Efficacy Scale TES.  Scales measured commitment to treatment orientation and self-efficacy (personal and general) | x |  |  |  | This study examined the correlation between commitment to specific treatment orientations and teacher self-efficacy | “Teachers who identified themselves with the ABA orientation had a significantly higher personal teaching self-efficacy compared to the TEACCH group, as well as the comparison group. No significant difference was found among the three groups in terms of general teaching self-efficacy.” | *Not explicitly stated. Both primary and possible lower secondary levels. | x | |  |
| 48 | Snyman, et al. (2023) | South Africa |  |  | x | 26 participant, all female educators | x |  | Pre- and post-intervention surveys  Weekly behavior tracking during the 6-week intervention  Scales:  SBTCC: 7-point frequency scale and 5-point severity scale for behaviors  DBSE: 7-point Likert scale  MHC-SF: 6-point scale (0 = never to 5 = every day) for well-being  Post-intervention self-report feedback used a 5-point Likert scale | x |  |  |  | To determine the effect of a strength-based intervention on educators’ perception of their own well-being, self-efficacy and the behaviour of autistic learners in their class | "The autistic learners showed a significant decrease in verbal aggression both in frequency and severity and in the severity of physical aggression, disruption, destruction and manipulative, deceitful or non-compliant behaviour. Educators evaluated the outcome of the intervention as salutary for the learners and participants." |  | x | |  |
| 49 | Stošić, (et al. 2022) | Croatia | x* |  | x** | 99 primary school teachers  95.9% female | x |  | Method: Structured questionnaire (anonymous, pilot-tested)  Scales (non-validated):  True/False for autism knowledge  Agreement statements for attitudes (e.g., Likert-type: Yes/No)  Categorical responses for training, confidence, and use of strategies | x |  |  |  | “…to investigate such teachers’ knowledge and attitudes regarding autism, and their experience of ‘good autism practice’ methods and approaches. | “This study has shown Croatian teachers’ knowledge to be inconsistent and often inaccurate, with ambivalent attitudes held towards the inclusion of learners with autism and low confidence regarding practice.” | *(32%)  **(68%) | x | |  |
| 50 | Taliaferro & Harris, (2014) | USA | x |  |  | N = 65 physical education teachers  Treatment group: 38 teachers from Maryland (15 elementary, 11 middle, 12 high school)  Control group: 27 teachers from other states (e.g., Virginia, New Jersey, California) | x | x | Pretest and post-test surveys using the PESEISD-A instrument.  Measures teachers’ self-efficacy toward 10 inclusion-related tasks | x |  |  |  | The purpose of this study was to determine the effects of a one-day workshop on the self-efficacy of general physical educators to include students with autism into the general physical education setting.” | “While the change in self-efficacy scores due to the treatment was not statistically significant, an increase of nearly one point was observed in teacher self-efficacy scores in the treatment group... This increase is promising and may have practical significance.” |  | x | |  |
| 51 | Van Mieghem, et al (2022) | Belgium | x |  |  | N = 692 teachers  After dropout: n = 610 completed background info  about 80% female | x | x | Online survey  Student-specific TSE scale (Zee & Koomen, 2015)  And a non-validated scale of nine support sources. | x |  |  |  | “This study has been undertaken to examine TSE regarding teaching students with SEN and how these beliefs relate to the sources of support available for them when teaching such students.” | We assume that making these forms of support more available to teachers will make them more competent to deal with students with SEN and will, therefore, reduce resistance to a more IE system.” (p. 39)  “We found that the more Cooperative sources of support were used… the higher the overall levels of student-specific self-efficacy.” | 59 primary and 61 secondary schools | x | |  |
| 52 | Wangsgard & Cardon, (2018) | USA | x |  |  | 10 general education teachers | x | x | Modified Teachers’ Sense of Efficacy Scale (TSES) (quantitative)  Focus group interview (qualitative) |  |  | x |  | To investigate general education teachers’ perceptions of their ability to meet the needs of students with autism spectrum disorder (ASD), focusing on their self-efficacy in instruction, classroom management, and student engagement. | Teachers felt confident in instructional practices and classroom management  Lowest confidence was in student engagement with ASD students  Qualitative findings revealed:  Lack of understanding of ASD-specific needs  Desire for more training, support, and smaller class sizes  Teachers rely on general strategies, not ASD-specific evidence-based practices | MMAT assessed as quantitative descriptive. | x | |  |
| 53 | Wearmouth & Butler (2020) | England | x* |  |  | 18 Special Educational Needs Coordinators (SENCos) | x | x | Electronic questionnaires and telephone interviews.  No validated questionnaires:  Likert-type scales (e.g., 6-point scales) were used for questions about:  Confidence levels  Staff willingness |  |  |  | x | The study aimed to explore:  How well SENCos and classroom teachers understand autism  Their ability to assess autistic pupils’ needs  Their capacity to adapt teaching and support inclusion | Inclusion efforts often fall short due to limited training, funding, and systemic support—despite legal obligations and good intentions. | *Mainly 1  9 in infant/lower schools, 3 in middle schools (ages 9–13), 3 in secondary schools  MMAT assessed as qualitative. |  | |  |
| 54 | Wittwer et al. 2024) | Germany | x |  |  | 887 teachers (726 females, 123 males, 38 no gender indicated) | x | x | Questionnaires: Autism Stigma and Knowledge Questionnaire (ASK-Q) + The Self-Efficacy for Autism Scale (TSEAS) developed by Love (2016) + Autism Self-Efficacy Scale for Teachers (ASSET) developed by Ruble et al. (2013) +  Autism Attitude Scale for Teachers (AAST) developed by Olley et al. (1981)  (adapted versions) | x |  |  |  | Explore knowledge, self-efficacy, and attitude concerning autism | Results indicated moderate teacher knowledge about autism, with some common misconceptions present. Although teachers displayed moderately positive attitudes toward inclusion, their self-efficacy beliefs were not particularly high. Experience teaching autistic students correlated with increased knowledge and self-efficacy, and female teachers exhibited higher knowledge and self-efficacy than male teachers. However, school type had minimal impact on teachers' knowledge, self-efficacy, and attitudes. |  | x | |  |
| 55 | Xie et al. 2024 | China | x |  |  | 972 teachers (16.8% male) | x |  | Questionnaire:  Teachers’ ATIE were measured using the Chinese version of the Multidimensional Attitudes Toward Inclusive Education Scale (MATIES; Xie & Zhang, 2022) + The Teacher Efficacy for Inclusive Practice (TEIP) scale was developed by Sharma et al. (2012) + The Social Support of Inclusive Education Teachers Question +The Principals’ Leadership Questionnaire | x |  |  |  | examine the effects of three school factors (i.e., school support, principals’ leadership, and in-service training) on attitudes towards inclusive education (ATIE) and the mediating role of self-efficacy in the relationships among teachers of students with developmental disabilities | Administrative support, emotional support, transformational leadership, and in-service training positively influence teachers' attitudes toward inclusive education (ATIE) for students with developmental disabilities, with teacher self-efficacy mediating this relationship. | Developmental disabilities “such as autism” | x | |  |
| 56 | Zappalà & Aiello  2023 | Italy | x |  |  | 275 teachers, response rate 49,60% = final sample 124 teacher (102 females and 22 males) | x |  | Google-Form semi-structured questionnaire |  | x |  |  | investigate future support teachers' views on their role in facilitating full participation of students with autism, and to examine whether attending the Specialization Course for Educational Support Activities for Pupils with Disabilities influences these views. | attending the course affected students’ opinions on the inclusion of students with autism due to the acquisition of a deeper knowledge on inclusive teaching | The participants were teachers and participated in a course at a university. Yes, since already teachers. | x | |  |
| 57 | Öhlböck, et al.  2024 | UK | x |  |  | 128 teachers completing outcome measures at timepoint 1 and attending the online training. At timepoint 2, completion dropped to 87 teachers for the TSES and 89 for the URP-IR. Timepoint 3, participation further decreased, with 41 teachers completing the TSES, and a complete dataset was ultimately obtained from **37** teachers. | x |  | Short form of the teachers’ sense of efficacy scale (TSE) & Usage rating profile-intervention revised (URP-IR) + qualitative survey asked open-ended questions to explore participants' experience |  |  | x |  | Explore the effect of an online training programme covering the main paradigms and resources of the Zones of Regulation™ curriculum on teachers' sense of self-efficacy when managing autistic students' self-regulation | The study found that online training in the Zones of Regulation™ curriculum significantly improved teachers' TSES scores (p < 0.001). Additionally, the curriculum was considered acceptable, understandable, and feasible for primary school teachers in mainstream classrooms. | MMAT assessed as quantitative nonrandomized. | x | |  |

^a^ 1=regular school, 2= small class/ resourced provision, 3=special school

^b^1= 1=primary school (year up to 6), 2 =secondary school/high school (begins at year 7) (UK)

^c^1=Quantitative, 2= Qualitative, 3= Mixed Methods (authors describe that they used MM, and describes type of MM research design and integrates results), 4=combination (without, defining study as MM, description of type of MM, or integration the results)

^d^ 1= yes, 2= No

0=Not appreciable (NA)
