# Supplementary material for: A Systematic Research Review on Teachers’ Self-Efficacy in Educating Autistic Students
Source: Autism Dev Lang Impair. 2025 Oct 29;10:23969415251392318. doi: 10.1177/23969415251392318 (PMC12576102; doi:10.1177/23969415251392318)
Supplement: sj-docx-2-dli-10.1177_23969415251392318 - Supplemental material for A Systematic Research Review on Teachers’ Self-Efficacy in Educating Autistic Students [file sj-docx-2-dli-10.1177_23969415251392318.docx]

| No | Autor & year | All types | | Qualitative | | | | | Quantitative RCT | | | | | Quantitative nonrandomized | | | | | Quantitative  descriptive | | | | | Mixed  Methods | | | | | comment |
| --- | --- | --- | --- | --- | --- | --- | --- | --- | --- | --- | --- | --- | --- | --- | --- | --- | --- | --- | --- | --- | --- | --- | --- | --- | --- | --- | --- | --- | --- |
|  |  | S1 | S2 | 1.1 | 1.2 | 1.3 | 1.4 | 1.5 | 2.1 | 2.2 | 2.3 | 2.4 | 2.5 | 3.1 | 3.2 | 3.3 | 3.4 | 3.5 | 4.1 | 4.2 | 4.3 | 4.4 | 4.5 | 5.1 | 5.2 | 5.3 | 5.4 | 5.5 |  |
| 1 | Accardo & Finnegan  (2017) | A | A |  |  |  |  |  |  |  |  |  |  |  |  |  |  |  | A | A | A | B* | A |  |  |  |  |  | 4.4 is reported as a limitation |
| 2 | Alallawi et al.  (2022) | B* | A | A | B* | A | A | A |  |  |  |  |  |  |  |  |  |  |  |  |  |  |  |  |  |  |  |  | S1, no RQ, clear aim.  1.2 10 in total |
| 3 | Alhumaid (2023) | B* | A |  |  |  |  |  |  |  |  |  |  |  |  |  |  |  | A | B | A | B* | A |  |  |  |  |  | S1, no clear RQ: s, but clear aim  4.4 reported as a limitation |
| 4 | Alkeraida (2023) | B* | A | A | A* | A | C | A |  |  |  |  |  |  |  |  |  |  |  |  |  |  |  |  |  |  |  |  | S1, no clear RQ: s, but clear aim  1.2 4 teachers |
| 5 | Anglim et al. (2018) | A | A | A | A | A | A | A |  |  |  |  |  |  |  |  |  |  |  |  |  |  |  |  |  |  |  |  |  |
| 6 | Baek et al. (2024) | A | A |  |  |  |  |  |  |  |  |  |  |  |  |  |  |  | A | A | A | B* | A |  |  |  |  |  | 4.4, reported as a limitation |
| 7 | Bertuccio (2019) | A | A |  |  |  |  |  |  |  |  |  |  | B* | A | A | B* | A |  |  |  |  |  |  |  |  |  |  | 3.1 + 3.4 discussed in limitations |
| 8 | Bitska et al. (2017) | A | A |  |  |  |  |  |  |  |  |  |  | A | B | A | B | C |  |  |  |  |  |  |  |  |  |  |  |
| 9 | Bond et al. (2017) | B* | A |  |  |  |  |  |  |  |  |  |  |  |  |  |  |  | A | B | A | A | A |  |  |  |  |  | S1, no RQ, but clear aim. |
| 10 | Boujut et al. (2017) | A | A |  |  |  |  |  |  |  |  |  |  |  |  |  |  |  | A | A | A | A | A |  |  |  |  |  |  |
| 11 | Breeman et al. (2016) | A | A |  |  |  |  |  | A | B* | A | B* | A |  |  |  |  |  |  |  |  |  |  |  |  |  |  |  | 2.2baseline differences  2.4 No blinding. |
| 12 | Cappe et al. (2017) | B* | A |  |  |  |  |  |  |  |  |  |  |  |  |  |  |  | A | A | A | B* | A |  |  |  |  |  | S1, no RQ, but clear aim.  4.4 discussed as a limitation in discussion. |
| 13 | Cappe et al. (2021) | B* | A |  |  |  |  |  |  |  |  |  |  |  |  |  |  |  | A | A | A | B* | A |  |  |  |  |  | S1, no RQ, but clear aim.  4.4 discussed as a limitation in discussion. |
| 14 | Catalano et al. (2023) | A | A |  |  |  |  |  |  |  |  |  |  |  |  |  |  |  | A | B* | A | B* | A |  |  |  |  |  | 4.2 purposeful convenience sample, discussed as a limitation  4.4 recruitment method. Not discussed. |
| 15 | Cook & Ogden (2021) | B* | A | A | A | A | A | A |  |  |  |  |  |  |  |  |  |  |  |  |  |  |  |  |  |  |  |  | S1, no RQ, but clear aim. |
| 16 | Corona et al. (2017) | A | A |  |  |  |  |  |  |  |  |  |  | B | A | B* | B* | B* |  |  |  |  |  |  |  |  |  |  | 3.3 substantial attrition or missing data by the post-training follow-up  3.4 no discussion of confounders  3.5 deviations from intended intervention. Acknowledge and discussed. |
| 17 | Devi & Ganguly (2024) | A | A | A | A | A | A | A |  |  |  |  |  |  |  |  |  |  |  |  |  |  |  |  |  |  |  |  |  |
| 18 | Dille (2013) | A | A |  |  |  |  |  | A | A | A | B | A |  |  |  |  |  |  |  |  |  |  |  |  |  |  |  |  |
| 19 | Egan & Kenny (2022) | B* | A | A | A | B | B | B |  |  |  |  |  |  |  |  |  |  |  |  |  |  |  |  |  |  |  |  | S1, no RQ, but clear aim. |
| 20 | Emmons & Zager (2018) | A | A |  |  |  |  |  |  |  |  |  |  | C | A | A | B | A |  |  |  |  |  |  |  |  |  |  |  |
| 21 | Hinton et al. (2008) |  |  |  |  |  |  |  | A | A | A | B | A |  |  |  |  |  |  |  |  |  |  |  |  |  |  |  | 2.4 there was no blinding of the “assessors.” |
| 22 | Horan & Merrigan (2019) | A | A |  |  |  |  |  |  |  |  |  |  |  |  |  |  |  |  |  |  |  |  | A | B | B | A | A |  |
| 23 | Humphrey & Symes (2013) | A | A |  |  |  |  |  |  |  |  |  |  |  |  |  |  |  | A | B | A | A | A |  |  |  |  |  |  |
| 24 | Johnson et al. (2021) | A | A |  |  |  |  |  | A | A | A* | B | A |  |  |  |  |  |  |  |  |  |  |  |  |  |  |  | 2.3 only responses from teachers that took pre-,and post-test were included. 2.4 Blinding was not implemented |
| 25 | Kingsdorf et al. (2024) | A | A |  |  |  |  |  |  |  |  |  |  |  |  |  |  |  | A | B | A | B | A |  |  |  |  |  |  |
| 26 | Kisbu-Sakarya &Doenyas (2021). |  |  |  |  |  |  |  | A | B | A | B | A |  |  |  |  |  |  |  |  |  |  |  |  |  |  |  | 2.2 light baseline differences between the groups, 2.4 blinding of outcome assessment, |
| 27 | Latorre-Coscuellela (2022) | A | A |  |  |  |  |  |  |  |  |  |  |  |  |  |  |  | A | B* | A | C | A |  |  |  |  |  | 4.2 partially  4.4 response rate not reported |
| 28 | Latorre-Coscuellela (2023) | A | A |  |  |  |  |  |  |  |  |  |  |  |  |  |  |  | A | B* | A | C | A |  |  |  |  |  | 4.2 partially  4.4 response rate not reported |
| 29 | Lisak Šegota et al. 2022 | A | A |  |  |  |  |  |  |  |  |  |  |  |  |  |  |  | B* | B* | B* | B* | A |  |  |  |  |  | 4.1 partially  4.2 sample is skewed  4.3 partially  4.4 high non-response, no dropout analysis |
| 30 | Love et al. (2020) | A | A |  |  |  |  |  | B* | A | A | B* | A |  |  |  |  |  |  |  |  |  |  |  |  |  |  |  | 2.1 no detail on method  2.4 assessor blinding? |
| 31 | Love et al. (2019) | A | A |  |  |  |  |  |  |  |  |  |  |  |  |  |  |  | A | C | A | C* | A |  |  |  |  |  | 4.4 possible self-selection |
| 32 | Lu et al. (2020) | A | A |  |  |  |  |  |  |  |  |  |  |  |  |  |  |  | C | B | A | C | A |  |  |  |  |  |  |
| 33 | Maddox et al. (2013) | A | A |  |  |  |  |  |  |  |  |  |  |  |  |  |  |  | A | B | A | C* | A |  |  |  |  |  | 4.4 no dropout analysis |
| 34 | Nemcek et al. (2024) | A | A |  |  |  |  |  |  |  |  |  |  |  |  |  |  |  | C | B | A | C | A |  |  |  |  |  |  |
| 35 | Nolan et al. (2019 | A | A |  |  |  |  |  |  |  |  |  |  |  |  |  |  |  |  |  |  |  |  | A | A | A | B* | A | 5.4 partially |
| 36 | Oh et al. (2010) | A | A |  |  |  |  |  |  |  |  |  |  |  |  |  |  |  | A | B* | A | B* | A |  |  |  |  |  | 4.2 partially  4.4Selection bias |
| 37 | Park et al. (2019) | A | A |  |  |  |  |  |  |  |  |  |  |  |  |  |  |  |  |  |  |  |  | A | A | A | B* | A | 5.4 Partially |
| 38 | Parsons et al. (2016) | A | A |  |  |  |  |  |  |  |  |  |  |  |  |  |  |  | B | B | A | C | A |  |  |  |  |  |  |
| 39 | Rakap et al. (2018) | A | A |  |  |  |  |  |  |  |  |  |  |  |  |  |  |  | A | B* | A | B | A |  |  |  |  |  | 4.2 low response rate |
| 40 | Rakap et al. (2015) | A | A |  |  |  |  |  |  |  |  |  |  | A | A | A | B* | A |  |  |  |  |  |  |  |  |  |  | 3.4 partially |
| 41 | Rodden et al. (2019) | A | A | A | A | A | A | A |  |  |  |  |  |  |  |  |  |  |  |  |  |  |  |  |  |  |  |  |  |
| 42 | Ruble et al. (2013) | A | A |  |  |  |  |  |  |  |  |  |  |  |  |  |  |  | A | B* | A | C | A |  |  |  |  |  | 4.2 partially |
| 43 | Ruble et al. (2011) | A | A |  |  |  |  |  |  |  |  |  |  |  |  |  |  |  | A | B* | A | C | A |  |  |  |  |  | 4.2 partially |
| 44 | Ryan & Mathews (2022a) | A | A |  |  |  |  |  |  |  |  |  |  |  |  |  |  |  | C | B | A | C | A |  |  |  |  |  | 4.4 response rate not reported |
| 45 | Ryan &Mathews (2022b) | A | A |  |  |  |  |  |  |  |  |  |  |  |  |  |  |  | C* | B | A | C* | A |  |  |  |  |  | 4.1 self-selection bias?  4.4 no nonresponse analysis |
| 46 | Selvaganapathi et al. (2019) | A | A |  |  |  |  |  |  |  |  |  |  |  |  |  |  |  | B | B | A | C | A |  |  |  |  |  |  |
| 47 | Siu & Ho (2010) | A | A |  |  |  |  |  |  |  |  |  |  |  |  |  |  |  | C | B | A | C* | A |  |  |  |  |  | 4.4 60% response rate |
| 48 | Snyman et al. (2023) | A | A |  |  |  |  |  |  |  |  |  |  | B* | A | A | B | A |  |  |  |  |  |  |  |  |  |  | 3.1 partially |
| 49 | Stošic et al. (2022) | A | A |  |  |  |  |  |  |  |  |  |  |  |  |  |  |  | A | B* | A | C | A |  |  |  |  |  | 4.2 partially |
| 50 | Taliaferro & Harris (2014) | A | A |  |  |  |  |  |  |  |  |  |  | B | A | A | B | A |  |  |  |  |  |  |  |  |  |  |  |
| 51 | Van Mieghem et al. (2022) | A | A |  |  |  |  |  |  |  |  |  |  |  |  |  |  |  | A | B* | A | C | A |  |  |  |  |  | 4.2 partially |
| 52 | Wangsgard & Cardon (2018) | A | A |  |  |  |  |  |  |  |  |  |  |  |  |  |  |  | A | A | A | C | A |  |  |  |  |  |  |
| 53 | Wearmouth & Butler (2020) | B* | A | A | A | A | A | A |  |  |  |  |  |  |  |  |  |  |  |  |  |  |  |  |  |  |  |  | *S1 not clear RQ but a clear aim |
| 54 | Wittver et al. (2024) | A | A |  |  |  |  |  |  |  |  |  |  |  |  |  |  |  | B | B | A | C | A |  |  |  |  |  |  |
| 55 | Xie et al. (2024) | A | A |  |  |  |  |  |  |  |  |  |  |  |  |  |  |  | B | B | A | C* | A |  |  |  |  |  | 4.4. self-selection bias? |
| 56 | Zappala & Aiello (2023) | A | A | A | A | A | A | A |  |  |  |  |  |  |  |  |  |  |  |  |  |  |  |  |  |  |  |  |  |
| 57 | Öhlböck et al. (2024) | A | A |  |  |  |  |  |  |  |  |  |  | B | A | B* | B | A |  |  |  |  |  |  |  |  |  |  | 3.3 large attrition |
