# Supplementary material for: A Systematic Research Review on Teachers’ Self-Efficacy in Educating Autistic Students
Source: Autism Dev Lang Impair. 2025 Oct 29;10:23969415251392318. doi: 10.1177/23969415251392318 (PMC12576102; doi:10.1177/23969415251392318)
Supplement: sj-docx-3-dli-10.1177_23969415251392318 - Supplemental material for A Systematic Research Review on Teachers’ Self-Efficacy in Educating Autistic Students [file sj-docx-3-dli-10.1177_23969415251392318.docx]

| # | First author/year | Analytical Categories |
| --- | --- | --- |
|  |  |  |
| 1 | Accardo & Finnegan (2017) | General profiles and patterns of teacher self-efficacy |
| 2 | Alallawi et al. (2022) | General profiles and patterns of teacher self-efficacy |
| 3 | Alhumaid (2021) | Teachers’ self-efficacy related to inclusion and participation |
| 4 | Alkeraida (2023) | Teachers’ self-efficacy related to inclusion and participation |
| 5 | Anglim et al. (2017) | General profiles and patterns of teacher self-efficacy |
| 6 | Baek et al. (2024) | General profiles and patterns of teacher self-efficacy |
| 7 | Bertuccio et al. (2019) | Professional development and targeted training in relation to teacher self-efficacy |
| 8 | Bitska et al. (2017) | Professional development and targeted training in relation to teacher self-efficacy |
| 9 | Bond et al. (2017) | Professional development and targeted training in relation to teacher self-efficacy |
| 10 | Boujut et al. (2017) | Self-efficacy, stress, and burnout |
| 11 | Breeman et al. (2016) | Professional development and targeted training in relation to teacher self-efficacy |
| 12 | Cappe et al. (2017) | Self-efficacy, stress, and burnout |
| 13 | Cappe et al. (2021) | General profiles and patterns of teacher self-efficacy |
| 14 | Catalano et al. (2023) | Development and validation of self-efficacy scales |
| 15 | Cook & Ogden (2021) | General profiles and patterns of teacher self-efficacy |
| 16 | Corona et al. (2017) | Professional development and targeted training in relation to teacher self-efficacy |
| 17 | Devi & Ganguly (2024) | Teachers’ self-efficacy related to inclusion and participation |
| 18 | Dille (2013) | Professional development and targeted training in relation to teacher self-efficacy |
| 19 | Egan & Kenny (2022) | General profiles and patterns of teacher self-efficacy |
| 20 | Emmons & Zager (2018) | Professional development and targeted training in relation to teacher self-efficacy |
| 21 | Hinton et al. (2008) | Professional development and targeted training in relation to teacher self-efficacy |
| 22 | Horan & Merrigan (2019) | Professional development and targeted training in relation to teacher self-efficacy |
| 23 | Humphrey & Symes (2013) | Teachers’ self-efficacy related to inclusion and participation |
| 24 | Johnson et al. (2021) | Professional development and targeted training in relation to teacher self-efficacy |
| 25 | Kingsdorf et al. (2024) | General profiles and patterns of teacher self-efficacy |
| 26 | Kisbu-Sakarya & Doenyas (2021) | Professional development and targeted training in relation to teacher self-efficacy |
| 27 | Latorre-Cosculluela et al. (2022) | Teachers’ self-efficacy related to inclusion and participation |
| 28 | Latorre-Cosculluet al. (2023) | Teachers’ self-efficacy related to inclusion and participation |
| 29 | Lisak Šegota, et al. (2022) | General profiles and patterns of teacher self-efficacy |
| 30 | Love et al. (2020) | Self-efficacy, stress, and burnout |
| 31 | Love et al. (2019) | Development and validation of self-efficacy scales |
| 32 | Lu et al. (2020) | General profiles and patterns of teacher self-efficacy |
| 33 | Maddox & Marvin (2013) | Professional development and targeted training in relation to teacher self-efficacy |
| 34 | Nemček et al. (2024) | Teachers’ self-efficacy related to inclusion and participation |
| 35 | Nolan & Hannah, (2019) | Professional development and targeted training in relation to teacher self-efficacy |
| 36 | Oh & Kozub (2010) | Development and validation of self-efficacy scales |
| 37 | Park, et al. (2019) | Professional development and targeted training in relation to teacher self-efficacy |
| 38 | Parsons et al. (2016) | Professional development and targeted training in relation to teacher self-efficacy |
| 39 | Rakap et al. (2018) | General profiles and patterns of teacher self-efficacy |
| 40 | Rakap et al. (2015) | Professional development and targeted training in relation to teacher self-efficacy |
| 41 | Rodden et al. (2019) | General profiles and patterns of teacher self-efficacy |
| 42 | Ruble et al. (2013) | Development and validation of self-efficacy scales |
| 43 | Ruble et al. (2011) | Self-efficacy, stress, and burnout |
| 44 | Ryan & Mathews (2022a) | General profiles and patterns of teacher self-efficacy |
| 45 | Ryan & Mathews (2022b) | General profiles and patterns of teacher self-efficacy |
| 46 | Selvaganapathi et al. (2019) | General profiles and patterns of teacher self-efficacy |
| 47 | Siu & Ho (2010) | General profiles and patterns of teacher self-efficacy |
| 48 | Snyman et al. (2023) | General profiles and patterns of teacher self-efficacy |
| 49 | Stošić et al. (2022) | General profiles and patterns of teacher self-efficacy |
| 50 | Taliaferro & Harris (2014) | Professional development and targeted training in relation to teacher self-efficacy |
| 51 | Van Mieghem et al. (2022) | General profiles and patterns of teacher self-efficacy |
| 52 | Wangsgard & Cardon (2018) | General profiles and patterns of teacher self-efficacy |
| 53 | Wearmouth & Butler (2020) | General profiles and patterns of teacher self-efficacy |
| 54 | Wittwer et al. (2024) | General profiles and patterns of teacher self-efficacy |
| 55 | Xie et al. (2024) | Teachers’ self-efficacy related to inclusion and participation |
| 56 | Zappalà & Aiello (2023) | Professional development and targeted training in relation to teacher self-efficacy |
| 57 | Öhlböck, et al. (2024) | Professional development and targeted training in relation to teacher self-efficacy |
